# Plasma pre-treatment and peroxynitric acid soaking as a novel oxidative seed disinfection strategy against seed-borne fungal pathogens in maize

**DOI:** 10.3389/fmicb.2026.1927228

**Published:** 2026-09-28

**Authors:** Nina Recek, Jure Mravlje, Rok Zaplotnik, Jernej Ekar, Mia Jarc, Katarina Vogel Mikuš, Peter Gselman, Satoshi Ikawa, Katsuhisa Kitano, Miran Mozetič

**Affiliations:** 1 Department of Surface Engineering, Jožef Stefan Institute, Ljubljana, Slovenia; 2 Department of Biology, Biotechnical Faculty, University of Ljubljana, Ljubljana, Slovenia; 3 Department of Low and Medium Energy Physics, Jožef Stefan Institute, Ljubljana, Slovenia; 4 Interkorn Ltd., Beltinci, Slovenia; 5 Osaka Research Institute of Industrial Science and Technology, Osaka, Japan; 6 Graduate School of Engineering, The University of Osaka, Osaka, Japan

**Keywords:** *Aspergillus flavus*, *Fusarium fujikuroi*, non-equilibrium plasma treatment, peroxynitric acid, seed disinfection, seed-borne fungal pathogens, sustainable agriculture

## Abstract

**Introduction:**

Seed-borne fungal contamination is an important challenge in maize production and may contribute to grain deterioration and mycotoxin contamination. This study evaluated peroxynitric acid (PNA), alone and in combination with nonequilibrium gaseous plasma pretreatment, as a rapid oxidative strategy for disinfecting maize seeds contaminated with *Aspergillus flavus* and *Fusarium fujikuroi*.

**Methods:**

Maize seeds were artificially inoculated with approximately 10⁶ spores of either *A. flavus* or *F. fujikuroi.* For plasma pretreatment, seeds were sequentially exposed to hydrogen (0.2 s) and oxygen (0.2 s) radio-frequency plasmas in a low-pressure reactor. Hydrogen plasma was sustained in H mode at 30 Pa and 800 W, whereas oxygen plasma was sustained in E mode at 50 Pa and 300 W. Seeds were subsequently exposed to 1 -400 mM PNA for 3 -300 s. Antifungal efficacy was assessed using direct plating assays and four-parameter logistic modeling to estimate the 50% and 90% inhibitory concentrations (IC50 and IC90) and effective exposure times (ET50 and ET90). Seed viability was evaluated by germination assays.

**Results:**

PNA exhibited rapid, concentration- and time-dependent antifungal activity against both fungal species. Strong suppression of *F. fujikuroi* infection was observed at PNA concentrations ≥100 mM, with complete suppression of detectable infection at 400 mM after 180 s. At 50 mM PNA, *A. flavus* showed substantially faster inactivation kinetics than F. fujikuroi, with estimated ET50 values of 42.3 and 95.1 s, respectively. At 100 mM PNA, plasma pretreatment shifted the fitted time -response curves toward shorter exposure times and lower infection levels, particularly for A. flavus. However, planned plasma-versus-no-plasma comparisons were not statistically significant after adjustment for overdispersion. Neither PNA nor plasma treatment significantly affected seed germination.

**Discussion:**

PNA demonstrated rapid and effective antifungal activity against seed-borne *A. flavus* and *F. fujikuroi*, with clear species-specific differences in susceptibility. Plasma pretreatment appeared to accelerate early PNA-mediated fungal suppression but did not consistently improve final disinfection efficacy at prolonged exposure times. This effect may be associated with increased seed-surface hydrophilicity following plasma exposure. The results support PNA as a promising oxidative approach for maize seed disinfection and provide further insight into species-specific responses and plasma-associated kinetic effects in seed-borne filamentous fungi.

## Graphical abstract

**Figure:**
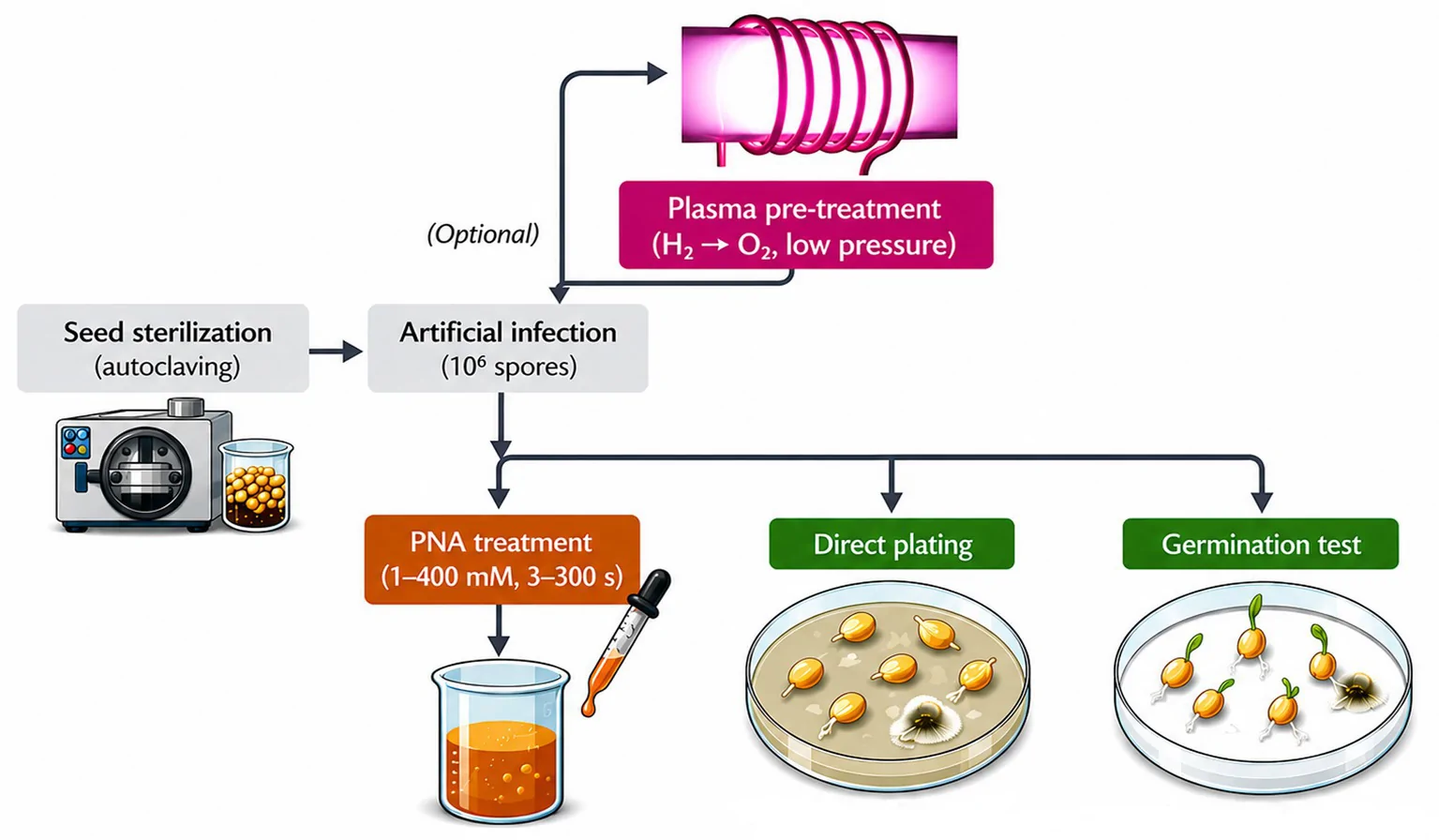
Schematic overview of the experimental design.

## 1 Introduction

Fungal contamination of agricultural commodities poses a major global challenge, significantly affecting crop yields, food safety, and the economic viability of crop production (Anand and Rajeshkumar, 2022; Singh and Thakur, 2023). This challenge is further exacerbated by the increasing spread and impact of disease-causing fungi, driven by human activities such as globalization, which facilitate the long-distance dispersal of fungal propagules, alongside ongoing environmental and climate change (Fisher et al., 2016). Filamentous fungi are widespread and can colonize cereal grains either in the field before harvest or during storage after harvest (Christensen, 1957). Seed-borne fungi are particularly important because they can compromise seed quality, cause disease in emerging seedlings, and contribute to losses throughout the production and storage chain. In maize, the combined preharvest grain yield losses, mycotoxin contamination, and storage losses associated with toxigenic fungi have been estimated at approximately 220–265 million metric tons (Mesterhazy, 2024). Fungal contamination of maize therefore has implications beyond yield reduction, as infection by toxigenic fungi can result in mycotoxin contamination, commodity downgrading or rejection, and substantial economic losses throughout the food and feed supply chain (Mitchell et al., 2016; Mesterhazy, 2024; Yohannis et al., 2025).

Among the most problematic fungi associated with cereal grains are *A. flavus* and *F. fujikuroi*, both of which can infect seeds and produce mycotoxins that pose serious risks to human and animal health (Fung and Clark, 2004). *A. flavus* is well known for its production of aflatoxins, which are among the most potent naturally occurring carcinogens (Klich, 2007). Members of the *F. fujikuroi* species complex, associated with diseases such as bakanae in rice, are also capable of colonizing maize and other crops (Placinta et al., 1999; Awuchi et al., 2021) and may produce several mycotoxins, including fumonisins (Munkvold, 2016). Effective control of seed-borne fungi is therefore essential not only for maintaining seed quality and germination but also for reducing the risk of subsequent crop infection and mycotoxin contamination.

Chemical fungicide seed treatment remains one of the most widely adopted and established approaches for controlling seed- and soil-borne fungal pathogens and improving seed emergence and early plant vigor (Mancini and Romanazzi, 2014; Ayesha et al., 2021). However, the extensive use of synthetic fungicides presents several limitations, including the persistence of chemical residues in agricultural products, adverse environmental impacts, and potential effects on human health (Sharma et al., 2019). Repeated application can also contribute to the selection and spread of fungicide-resistant fungal populations (Gupta and Kumar, 2020; Lamichhane et al., 2020). Moreover, chemical seed treatments may adversely affect seed viability and germination and may disrupt the seed-associated microbiome and beneficial endophytes (Singh et al., 2020; Ayesha et al., 2021). These concerns have stimulated increasing interest in alternative decontamination strategies that are effective against fungal pathogens while minimizing chemical inputs and adverse effects on seeds and the environment.

Non-equilibrium, or cold, plasma has emerged as a promising non-thermal technology for microbial decontamination (Moreau et al., 2008; Scholtz et al., 2021; Akpor et al., 2024). Plasma is a partially ionized gas containing a complex mixture of reactive and energetic species, including ions, electrons, ultraviolet photons, and neutral reactive species such as atoms, radicals, molecular fragments, and metastable electronically excited molecules. Cold plasma is particularly attractive for agricultural applications because it can inactivate microorganisms without exposing treated materials to the high temperatures associated with conventional thermal processes. Its antimicrobial activity is largely attributed to the generation of reactive oxygen and nitrogen species (RONS), which can damage essential cellular components and ultimately lead to microbial inactivation (Harikrishna et al., 2023). In recent years, plasma technologies have therefore been extensively investigated for applications in food safety, surface sterilization, and seed treatment (Bilea et al., 2024; Xu and Bassi, 2025). In addition to its direct antimicrobial effects, plasma can modify surface properties such as wettability and surface chemistry, potentially facilitating subsequent interactions between treated surfaces and liquid disinfectants (Zver et al., 2024; Holländer and Behnisch, 1998).

Plasma-treated liquids represent another promising approach because reactive species generated during plasma exposure can be transferred to, or retained within, the liquid phase (Ikawa et al., 2016; Rezaei et al., 2019). Among these reactive compounds, peroxynitric acid (PNA) has been identified as a highly reactive oxidant with strong antimicrobial potential (Yokoyama et al., 2021). PNA is formed through the reaction of hydrogen peroxide with nitrous acid and exhibits both oxidative and nitrative reactivity (Yokoyama et al., 2021). Importantly, PNA decomposes relatively rapidly into less persistent reaction products, making it an attractive candidate for applications where effective microbial inactivation is required while minimizing the persistence of chemical residues in the environment (Løgager and Sehested, 1993; Iwaki et al., 2020).

Recent studies have further highlighted the importance of the dynamic chemistry of plasma-generated RONS in determining antimicrobial efficacy. Pogoda et al. (2026) demonstrated that the bactericidal and sporicidal activity of plasma-treated aqueous solutions depends not only on the concentration of individual reactive species but also on their temporal evolution and the physicochemical properties of the treated liquid, including its buffering capacity. These findings suggest that antimicrobial activity should not be considered solely as a function of the initial concentration of a particular reactive species, but rather as the outcome of a dynamic network of chemical reactions occurring within the treatment environment. This consideration is particularly relevant to biological surfaces, where surface properties and local moisture conditions may influence the generation, transformation, transport, and persistence of reactive species (Pogoda et al., 2026).

The relevance of plasma-generated RONS to the control of plant-associated fungi has also been demonstrated. Watson et al. (2025) investigated cold atmospheric-pressure plasma treatment of liquid media for the control of *Pythium ultimum* and showed that plasma treatment substantially inhibited fungal/oomycete growth. The antimicrobial activity was associated with the formation of RONS and was influenced by the plasma configuration and gas composition, highlighting the importance of plasma–liquid interactions and the resulting chemical environment in determining treatment efficacy (Watson et al., 2025). More broadly, reviews of cold-plasma applications emphasize that microbial inactivation depends on the plasma configuration, treatment conditions, reactive species generated, and their interactions with the treated material (Cavalcanti et al., 2023). Collectively, these findings indicate that the antimicrobial effectiveness of non-thermal plasma is governed by a complex interplay between plasma-generated reactive species, the aqueous phase, the target microorganism, and the physicochemical characteristics of the treated surface.

In the context of maize seed disinfection, this dynamic perspective is particularly relevant because the seed surface and its associated moisture may provide a reaction environment in which plasma-generated RONS undergo further chemical transformations. Plasma pretreatment may therefore influence not only the direct inactivation of fungal contaminants but also the physicochemical properties of the seed surface and the subsequent interactions of reactive compounds with seed-associated microorganisms. Understanding these interactions is important for evaluating combined plasma and PNA treatments and for interpreting their potential synergistic or complementary effects.

Despite the growing understanding of plasma-generated RONS and the antimicrobial activity of PNA, the effects of PNA have been investigated predominantly in bacterial systems (Yokoyama et al., 2021), while its potential antifungal activity against filamentous fungi remains poorly understood. Furthermore, the potential of combining plasma pretreatment with subsequent PNA application for the disinfection of fungal-contaminated seeds has not been investigated. Given the ability of plasma to generate reactive species and modify surface properties, such a combined treatment may provide complementary mechanisms for fungal inactivation and enhance the interaction of PNA with contaminants on the seed surface.

The present study, therefore, aimed to evaluate the antifungal efficacy of PNA against *A. flavus* and *F. fujikuroi* on maize seeds and to determine whether plasma pretreatment enhances PNA-mediated disinfection. We hypothesized that PNA would exhibit significant antifungal activity against seed-borne *A. flavus* and *F. fujikuroi* and that plasma-induced surface activation would facilitate interactions between PNA and fungal contaminants on the seed surface, thereby improving the efficiency of fungal inactivation.

## 2 Methods

### 2.1 Experimental design

The antifungal efficacy of PNA applied either alone or after plasma pre-treatment was evaluated under controlled laboratory conditions. Maize seeds, hybrid P9241, were first sterilized by autoclaving and then artificially inoculated with spores at a defined concentration. Seeds were either left untreated by plasma or exposed to plasma pre-treatment, followed by chemically disinfected with PNA and microbiological direct plating to assess the proportion of seeds showing visible fungal growth. Germination tests were conducted in parallel on non-autoclaved seeds to evaluate treatment effects on seed viability.

### 2.2 Seed preparation and artificial infection

Commercial maize seeds were first sterilized by autoclaving to eliminate background microbial contamination. Following sterilization, seeds were artificially inoculated as described previously (Mravlje et al., 2023). Two fungal species were selected, namely *A.s flavus* (strain GB005) and *F. fujikuroi* (strain GB011). Both fungal strains used in these experiments were previously isolated, characterized, and deposited in the fungal bank of the Laboratory for Plant Physiology at the Department of Biology, Biotechnical Faculty, University of Ljubljana.

### 2.3 Plasma pre-treatment

Plasma pretreatment was performed in a low-pressure, RF plasma rotatable reactor (Figure 1) using a 13.56 MHz generator. High-purity hydrogen (99.999%) and oxygen (99.999%) were used as process gases. The plasma was generated under low-pressure conditions, with the operating pressure and RF power set to 30 Pa and 800 W for hydrogen plasma and 50 Pa and 300 W for oxygen plasma, respectively. During seed treatment, the reactor was positioned vertically, allowing seeds to free-fall through the plasma region to promote uniform exposure and surface treatment. The same reactor configuration and operating conditions were used for all experiments. In order to ensure reproducibility of the plasma generation, discharge parameters such as gas flow, pressure, forward and reflected RF power were carefully monitored and kept constant between experimental runs.

**Figure 1:**
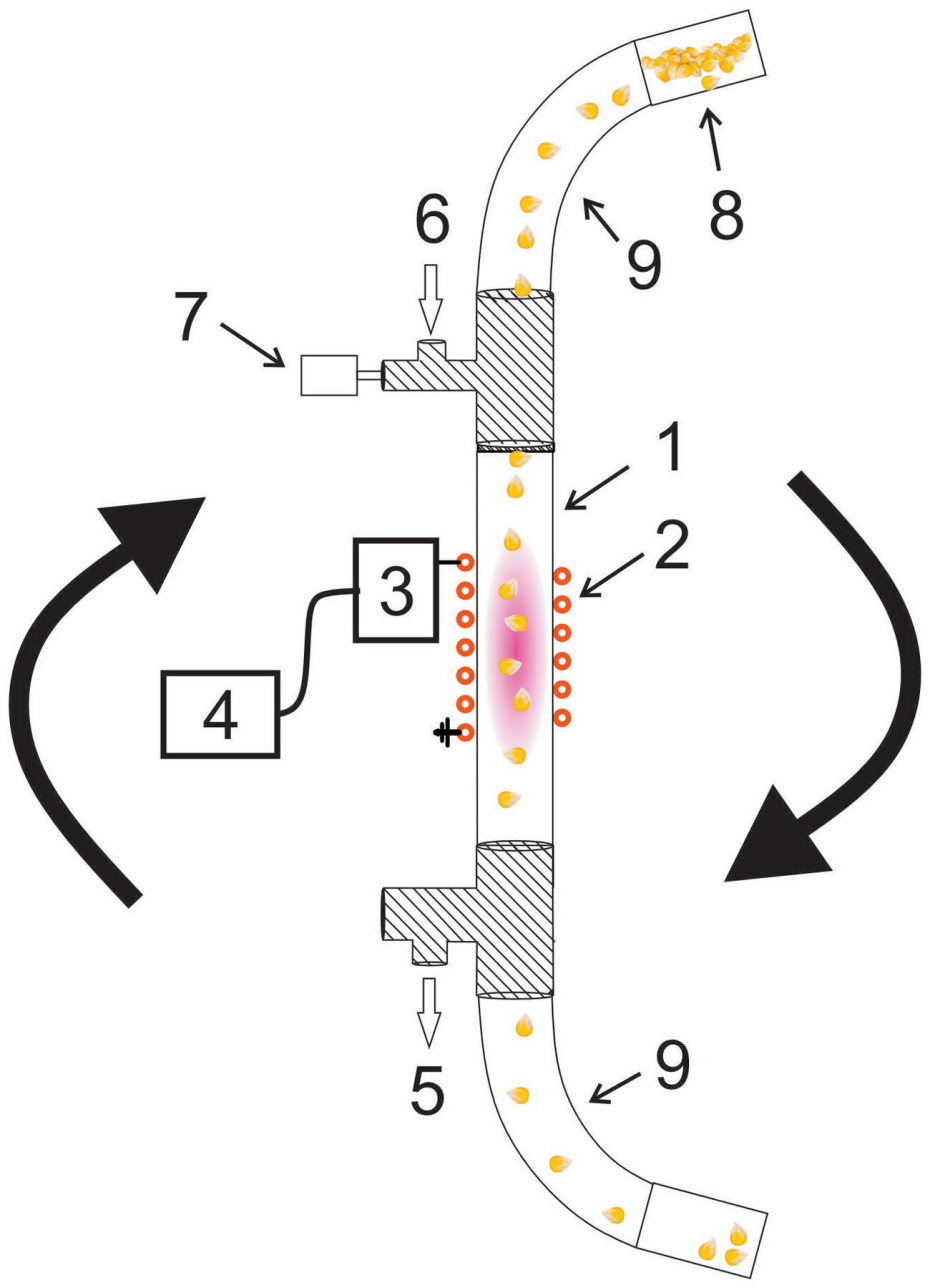
Rotatable experimental setup: 1 – the discharge tube, 2 – a copper coil, 3 – matching network, 4 – RF generator, 5 – pumping, 6 – gas inlet, 7 – pressure gauge, 8 – container for the seeds, 9 – flexible bellows.

For each treatment, seeds from the same lot were subjected to identical plasma exposure conditions and subsequently processed using the same PNA treatment protocol. Each experimental condition was performed using independent biological replicates, with untreated seeds included as controls. Plasma treatment, PNA treatment, and subsequent fungal recovery were performed using the same procedures across experimental replicates to ensure comparability between treatments.

A defined batch of maize seeds was placed in the sample container of the vertical system as indicated in Figure 1. The sample container was connected to the discharge tube via a flexible bellows connector. The system was then evacuated to the final pressure of about 2 Pa. The evacuation took about two minutes. After reaching the ultimate pressure, gas was introduced into the experimental system through the needle valve to achieve the desired working pressure. Then, the plasma was ignited, and the discharge tube was positioned vertically. The sample container was slowly lifted and shaken so that the seeds dropped through the plasma by free fall. The plasma treatment time per pass was thus about 0.2 s. After the seeds had passed through the plasma region, the discharge tube was turned around, and the RF generator was turned off. Such a simple procedure enabled very short and reproducible plasma exposure of the seeds. In the present study, each plasma stage consisted of a single pass through the discharge tube.

The plasma treatment of seeds was performed in two stages. The first stage consisted of a single pass through hydrogen plasma generated in H-mode at 30 Pa and a forward power of 800 W, followed by the second stage, which consisted of a single pass through oxygen plasma generated in E-mode at 50 Pa and 300 W. Thus, the total direct plasma exposure was approximately 0.4 s. The modes were chosen according to the desired plasma species. The E-mode oxygen plasma has a rather low electron density and high density of neutral O-atoms in the ground state, while the H-mode hydrogen plasma has a moderately large electron density, a high density of H atoms, and is a powerful source of VUV radiation (Primc et al., 2021; Fantz et al., 2016). The seeds remained under vacuum conditions between the two stages, preventing exposure to ambient air.

### 2.4 Synthesis and application of peroxynitric acid

PNA was synthesized using a low-temperature acidic–oxidative reaction system. A mixture of HNO_3_ (4 M, 17 mL), H_2_O_2_ (30%, 22 mL), and deionized water (14 mL) was cooled to approximately −5° C in a stainless steel container by immersion in a mixture of ethanol and isopropanol at −80° C. A solution of 40% sodium nitrate (NaNO_3_ → NaNO_2_) was slowly added at 1 mL min^−1^ to the HNO_3_-H_2_O_2_ mixture over 13 min. The reaction mixture was kept at a temperature between −5 °C and −2 °C and stirred vigorously during the addition of NaNO_2_ to prevent PNA decomposition. More details about the PNA synthesis can be found in the published literature (Yokoyama et al., 2020).

The concentration of PNA was colorimetrically determined using the DPD assay, which exploits the coloration of *N*, N-diethyl-p-phenylenediamine (DPD) upon its PNA-induced oxidation. The absorbance of the reddish-pink solution was measured at 553 nm. The DPD assay mixture was prepared by combining 200 mM sodium citrate buffer at pH 5 (35 μL), DPD solution at 50 g/L (20 μL), and deionized water (225 μL). To ensure measurements within the linear absorbance range, the PNA reaction mixture needed to be diluted 10,000-fold overall. In the first step, 50 μL of PNA reaction mixture was mixed with 950 μL of deionized water at 0 °C. In the second step, 30 μL of the PNA solution prepared in the first step was mixed with 970 μL of deionized water at 0 °C. Finally, 20 μL of the PNA solution prepared after the two dilution steps was added to 280 μL of the DPD assay mixture at room temperature and immediately stirred. Absorbance measurements were performed at 5-min intervals after PNA addition to the DPD assay mixture. This absorbance–concentration correlation had been previously determined by direct measurement of PNA concentration measurements with UV–Vis spectroscopy. Considering the total dilution factor, the original PNA stock concentration was calculated from the measured concentration after dilution. Pure PNA in water was isolated via ion-exchange chromatography, and its absorbance was measured between 200 and 290 nm (Nakashima et al., 2016).

For seed disinfection, 25 mL of 1 M sodium citrate buffer at pH 3.5, 50 μL of 100 mM ethylenediaminetetraacetic acid (EDTA) solution, the required volume of PNA reaction mixture containing NaNO_3_ and small amounts of excess H_2_O_2_, and the calculated volume of distilled water needed to reach the target final PNA concentration were added into separate sterile beakers. The PNA concentration in its reaction mixture was approximately 1 M. The amount of PNA added to the treatment solution was calculated from the exact PNA concentration and diluted accordingly, so that the PNA concentration in the seed-treatment solution ranged from 1 mM to 400 mM. For each experimental group, 15 g of maize seeds per experimental group were added to the sterilization mixture in beakers. The mixture was gently agitated to ensure homogeneity.

Prior to seed treatment, all necessary materials, including a timer, a magnetic stirrer where used, a sterile sieve, distilled water, and sterile plastic Petri dishes, were prepared to ensure precise timing during PNA exposure, which was the most critical step of the procedure. The calculated volume of PNA was then added to the beaker containing the seeds, and the timer was started simultaneously. The beaker was placed on a magnetic stirrer before PNA addition, and the solution was continuously stirred throughout the treatment.

After the designated exposure time (3–300 s), the contents of the beaker were poured through a sterile sieve and rinsed with sterile distilled water to stop the exposure and remove residual PNA. The treated seeds were then transferred into sterile plastic Petri dishes, sealed, and labeled. The same procedure was repeated for all treatment conditions (different PNA concentrations and different soaking times).

### 2.5 Microbiological analysis

The antifungal efficacy of PNA on maize seeds, with or without cold plasma pre-treatment was assessed using a direct plating assay. To assess fungal growth, the Ulster method (Muskett and Malone, 1941) was used. Treated seeds were placed directly on 2% potato dextrose agar (PDA) plates (90 mm in diameter) supplemented with the antibiotic chloramphenicol (50 mg L^−1^) to suppress bacterial growth, and incubated for one week at 24 °C in the dark. Five maize seeds were placed on each agar plate. For each treatment group, five replicates were prepared. Infection rates (IR) were calculated as the percentage of seeds exhibiting visible fungal colonies:


$$\mathrm{IR}\;(\%)=\frac{\text{number of infected seeds}}{\text{total muber of seeds}}\times 100$$

A seed was scored as infected when visible fungal growth developed from or around the seed after incubation.

### 2.6 Germination assay

To evaluate the impact of treatments on seed viability, germination tests were conducted using non-autoclaved seeds. Treated and untreated seeds were placed into Petri dishes (diameter 90 mm) on two layers of filter paper, moistened with 5 mL of sterile distilled water, and incubated in plant growth chambers at 24 °C, with 60% humidity, in the dark. For each treatment and control group, a germination assay was performed in triplicate with15 seeds in each Petri dish. Germination percentages (GP) were recorded after 7 days incubation. Seeds were scored as germinated when visible radicle emergence was observed. GP was calculated as follows:


$$\mathrm{GP}\;(\%)=\frac{\text{number of germinated seeds}}{\text{total number of seeds}}\times 100$$

### 2.7 Statistical analysis

All statistical analyses were performed using custom Python workflows developed for concentration–response, time–response, grouped plasma/PNA experiments, and germination testing. Full statistical outputs, model parameters, pairwise comparisons, effect-size estimates, and additional methodological details are provided in Supplementary Tables S1–S19 and the Supplementary methods.

For seed-infection assays, fungal infection was recorded as the number of infected seeds out of the total number of plated seeds per replicate. Although results are presented as percentages for clarity, statistical analyses were performed on the underlying infected and non-infected seed counts to preserve the binomial structure of the data (Agresti, 2013). Overall treatment effects were assessed using binomial models. Overdispersion was evaluated using the Pearson chi-square statistic divided by the residual degrees of freedom. When overdispersion was present, dispersion-adjusted inference was used rather than relying solely on nominal binomial-model *p*-values. Concentration–response and time–response relationships were characterized using four-parameter logistic (4PL) models (Ritz and Streibig, 2005; Ritz et al., 2015):


$$Y(x)=A_{0}+\frac{A_{\infty }-A_{0}}{1+{\left(\frac{x_{50}}{x}\right)}^{h}},$$

where (*Y(x)*) is the fitted response, (*x*) is the PNA concentration or exposure time, (*A_0_*) is the fitted response asymptote as (*x*) approaches zero, (*A_∞_*) is the fitted response asymptote at high (x), (x_50_) is the midpoint of the fitted response transition, and (*h*) is the Hill slope. The 4PL model was selected because the observed response patterns were consistent with monotonic sigmoidal relationships and because it permits independent estimation of the two asymptotes, the midpoint, and the steepness of the response transition. This was particularly relevant because complete fungal suppression was not reached within all experimentally tested ranges. Alternative nonlinear model families, such as Weibull or Gompertz models, were not formally compared because the 4PL model was selected *a priori* for estimation of the predefined treatment-response endpoints.

For concentration–response experiments, model-derived IC50 and IC90 values represent the PNA concentrations corresponding to 50 and 90%, respectively, of the fitted transition from (A_0_) toward (A_∞_). For time–response experiments, the corresponding model-derived effective times were reported as ET50 and ET90. These values correspond to the exposure times at which 50 and 90%, respectively, of the fitted response transition had occurred. Uncertainty in IC50, IC90, ET50, and ET90 estimates was quantified using percentile-based 95% bootstrap confidence intervals. Zero-concentration and zero-time controls were retained in the figures for reference but were excluded from 4PL fitting. ET90 estimates exceeding the maximum experimentally tested exposure time were considered model-based extrapolations and interpreted accordingly. Model convergence and descriptive goodness of fit were assessed as described in the Supplementary methods.

Pairwise comparisons of infection proportions were performed using two-proportion tests with Holm correction for multiple comparisons (Holm, 1979). When overdispersion was present, dispersion-adjusted *p*-values were used for inference. For experiments including plasma pre-treatment, separate 4PL time–response models were fitted to the PNA-only and plasma + PNA groups, and planned plasma-versus-no-plasma comparisons were performed at individual exposure times. Effect-size calculations are described in the Supplementary methods (Hedges, 1981).

For seed germination, normality and homogeneity of variance were assessed using Shapiro–Wilk and Levene tests, respectively. Because sample sizes were small and parametric assumptions were not consistently satisfied, treatment effects were evaluated using the Kruskal–Wallis test followed by pairwise Mann–Whitney U tests with Holm correction. Effect-size, biological-relevance, smallest effect size of interest (SESOI), and equivalence analyses are described in detail in the Supplementary methods (Hedges, 1981; Lakens, 2017).

## 3 Results

### 3.1 Effect of PNA concentration on fungal inactivation

Artificially inoculated maize seeds, without plasma pre-treatment, were exposed to PNA solutions for 180 s and analyzed microbiologically as described in Section 2.5. *F. fujikuroi* was selected for the concentration–response experiment because preliminary observations indicated greater tolerance to PNA than *A. flavus*. Infection remained 100% in the untreated control and in the 1 and 10 mM PNA groups, whereas infection decreased markedly at higher concentrations (Figure 2). Mean infection was 16% at 100 mM, 10% at 200 mM, 3.3% at 300 mM, and 0% at 400 mM, corresponding to 84, 90, 96.7, and 100% reductions relative to the untreated control, respectively.

**Figure 2:**
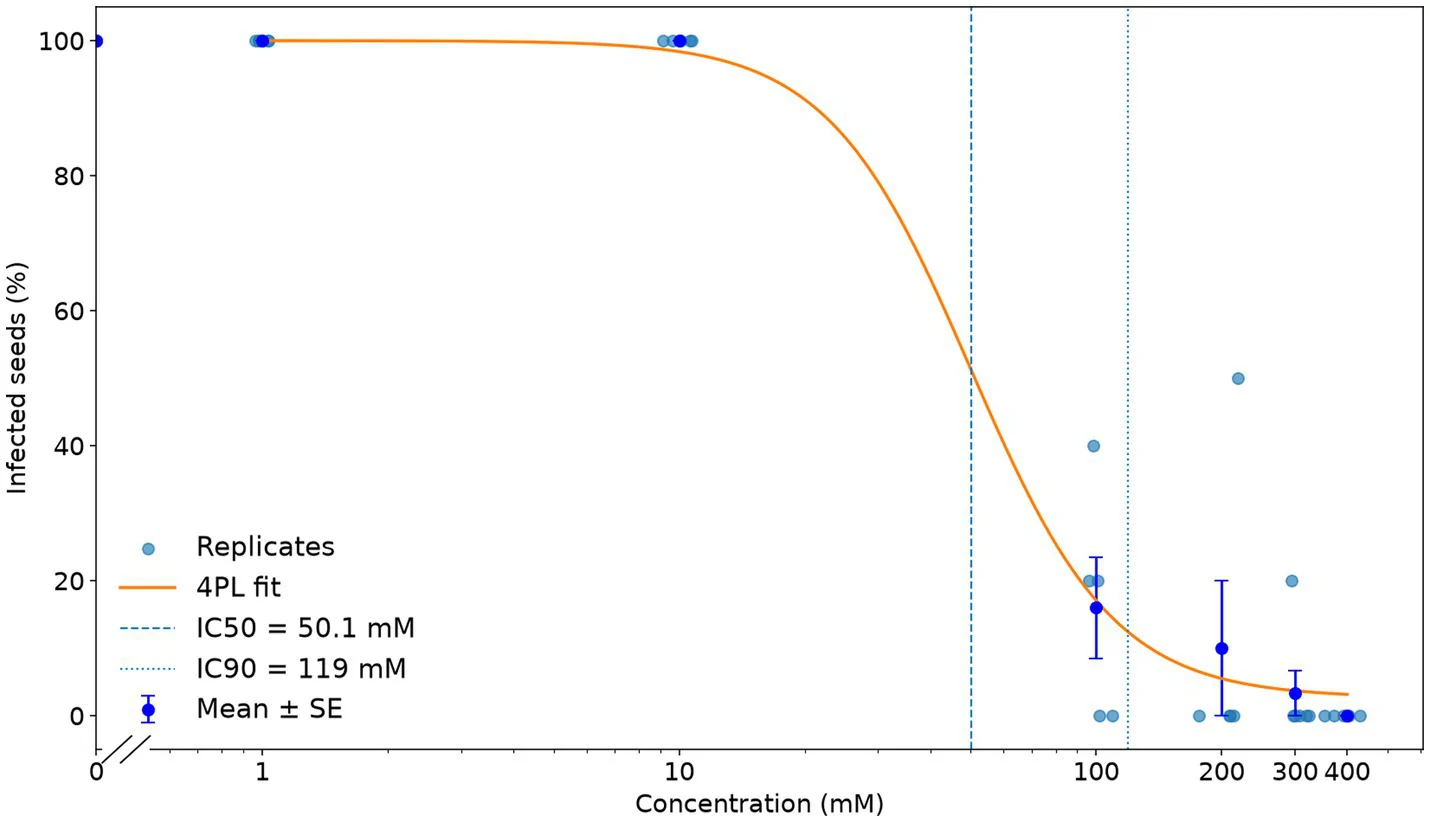
Concentration-dependent suppression of *F. fujikuroi* infection in artificially inoculated maize seeds after 180 s of exposure to PNA. Individual points represent replicate measurements (*n* = 5, except *n* = 6 at 300 mM), solid symbols show means ± SE, and the solid curve represents the fitted four-parameter logistic (4PL) model. Vertical dashed and dotted lines indicate the model-derived inhibitory concentration - IC_50_ and IC_90_, respectively. The x-axis is logarithmic; the 0 mM control is displayed at an offset position for visualization only and was not included in model fitting. For detailed statistical analysis, see Supplementary Tables S1–S3.

A four-parameter logistic (4PL) model described the concentration–response relationship. The estimated IC_50_ was 50.1 mM (95% bootstrap CI: 27.35–94.87 mM), while the IC_90_ was 119 mM (95% bootstrap CI: 30.61–209.1 mM). The relatively wide IC_90_ interval indicates uncertainty in the concentration required for 90% suppression. The overall effect of PNA concentration was statistically significant (χ^2^ = 3070.2, df = 6, *p* < 0.0001), although substantial overdispersion was present (Pearson χ^2^/df = 14.10). Detailed analyses are provided in Supplementary Tables S1–S3.

Under the tested 180 s exposure, 100 mM was the lowest concentration producing strong suppression of *F. fujikuroi* infection, whereas concentrations of 300–400 mM produced near-complete or complete suppression. These results define the concentration range used for subsequent time-dependent and plasma-pre-treatment experiments.

### 3.2 Species-dependent susceptibility to PNA

To compare the time-dependent susceptibility of the two fungal species, *F. fujikuroi* and *A. flavus*, a PNA concentration of 50 mM was selected. This concentration corresponds approximately to the IC_50_ of 50.1 mM estimated from the preceding concentration–response experiment with *F. fujikuroi* and therefore represents a suitable discriminatory concentration for comparing fungal sensitivity. A clear exposure-time-dependent reduction in seed infection was observed after treatment with 50 mM PNA (Figure 3). However, the response differed between the two fungal species, indicating species-specific susceptibility to PNA-mediated inactivation.

**Figure 3:**
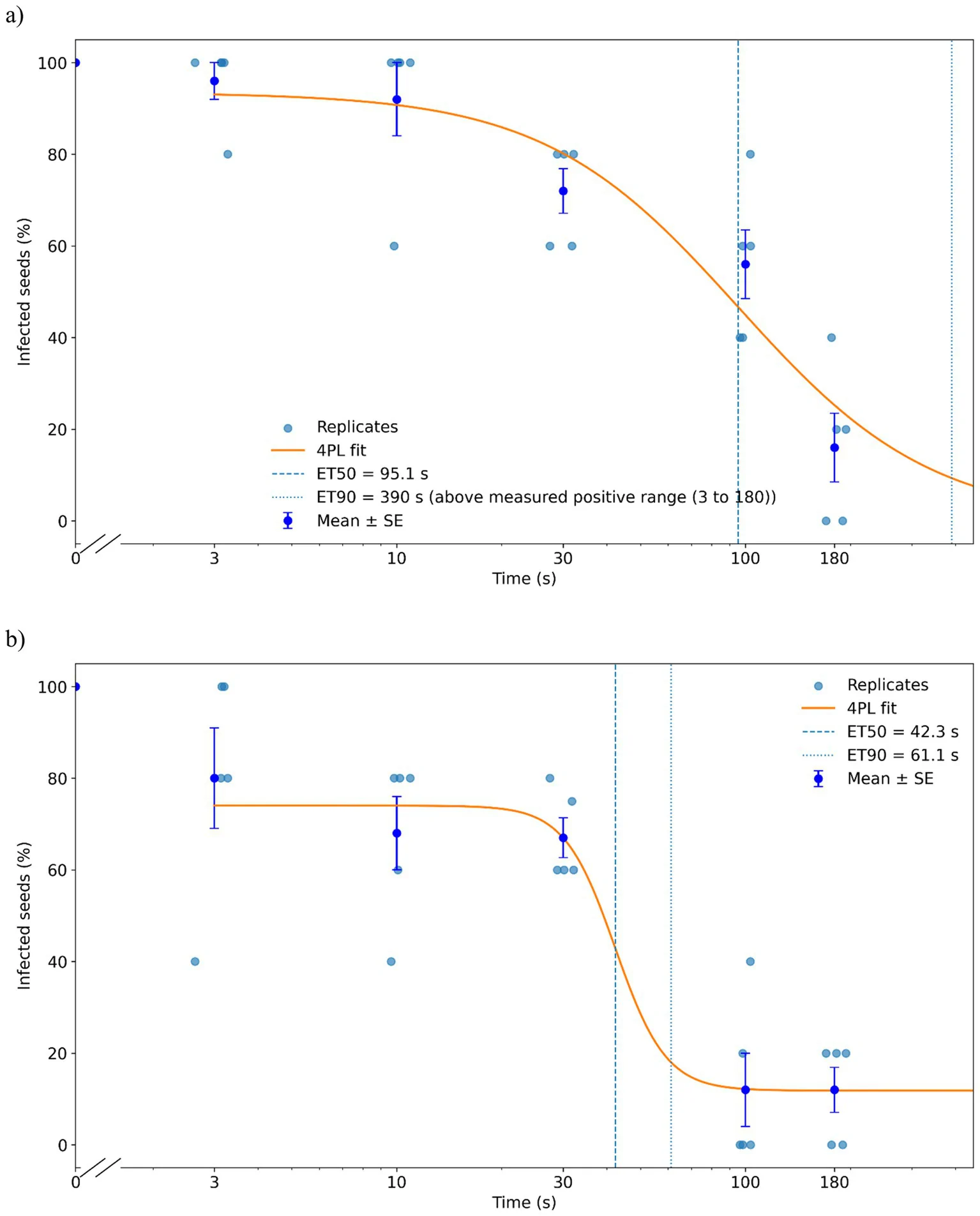
Time-dependent antifungal efficacy of 50 mM peroxynitric acid (PNA) against two fungal species inoculated onto maize seeds. **(a)** *F. fujikuroi* and **(b)** *A. flavus*. Individual points represent replicate measurements (*n* = 5), solid symbols show means ± SE, and solid curves represent fitted four-parameter logistic (4PL) models. Vertical dashed and dotted lines indicate the model-derived ET_50_ and ET_90_, respectively. The *x*-axis is logarithmic; 0 s controls are displayed at offset positions for visualization only and were not included in model fitting. Both panels are shown on the same x-axis scale to facilitate comparison between species. Effect of cold plasma pre-treatment on PNA-mediated fungal inactivation.

For *F. fujikuroi* (Figure 3a), mean infection was 96, 92, 72, 56, and 16% after 3, 10, 30, 100, and 180 s, respectively. The 4PL model estimated an ET_50_ of 95.1 s (95% bootstrap CI: 62.88–135.2 s) and an ET_90_ of 389.6 s (95% bootstrap CI: 158.9–767.6 s). Because the ET_90_ exceeded the longest experimental exposure, it represents a model-based extrapolation rather than a directly observed treatment time. The overall effect of exposure time was statistically significant (χ^2^ = 1277.8, df = 5, *p* < 0.0001), with substantial overdispersion (Pearson χ^2^/df = 17.08). At 180 s, infection was reduced by 84% but remained detectable. Thus, within the tested range, 50 mM PNA did not achieve 90% suppression of *F. fujikuroi*, and longer exposure or higher PNA concentrations would be required to approach this endpoint.

In contrast, *A. flavus* showed a faster response to 50 mM PNA (Figure 3b). Mean infection was approximately 80, 68, 68, 12, and 12% after 3, 10, 30, 100, and 180 s, respectively. The 4PL model estimated an ET_50_ of 42.3 s (95% bootstrap CI: 32.35–87.50 s) and an ET_90_ of 61.1 s (95% bootstrap CI: 37.29–298.8 s). The wide ET_90_ interval reflects uncertainty around the steep response transition. The overall effect of exposure time was statistically significant (χ^2^ = 1352.4, df = 5, *p* < 0.0001), with substantial overdispersion (Pearson χ^2^/df = 16.36).

The model estimates therefore indicated faster inactivation of *A. flavus* than *F. fujikuroi* at 50 mM PNA: ET_50_ values were approximately 42 and 95 s, respectively. The estimated ET_90_ was approximately 61 s for *A. flavus* and 390 s for *F. fujikuroi*, although the latter was extrapolated beyond the tested exposure range. These differences indicate species-specific inactivation kinetics under the tested conditions (Cohen, 2013).

To evaluate whether plasma pre-treatment was associated with altered PNA-mediated inactivation, inoculated seeds were exposed to cold plasma and subsequently treated with 100 mM PNA for increasing exposure times. The 100 mM concentration was selected because it produced strong suppression in the preceding concentration–response experiment. Plasma-versus-no-plasma comparisons were evaluated using the planned statistical analyses with adjustment for overdispersion.

In *F. fujikuroi* (Figure 4a), infection decreased progressively with increasing PNA exposure time in both treatment groups. Without plasma pre-treatment (Figure 4a), mean infection was 68, 64, 44, 36, and 28% after 3, 10, 30, 100, and 180 s, respectively, with complete suppression at 300 s. Following plasma pre-treatment, mean infection was 60, 56, 28, 28, and 24% at the corresponding exposure times, with complete suppression also observed at 300 s. Thus, lower mean infection was observed after plasma pre-treatment at most exposure times, particularly at 30 s, but both treatments produced the same final outcome at 300 s.

**Figure 4:**
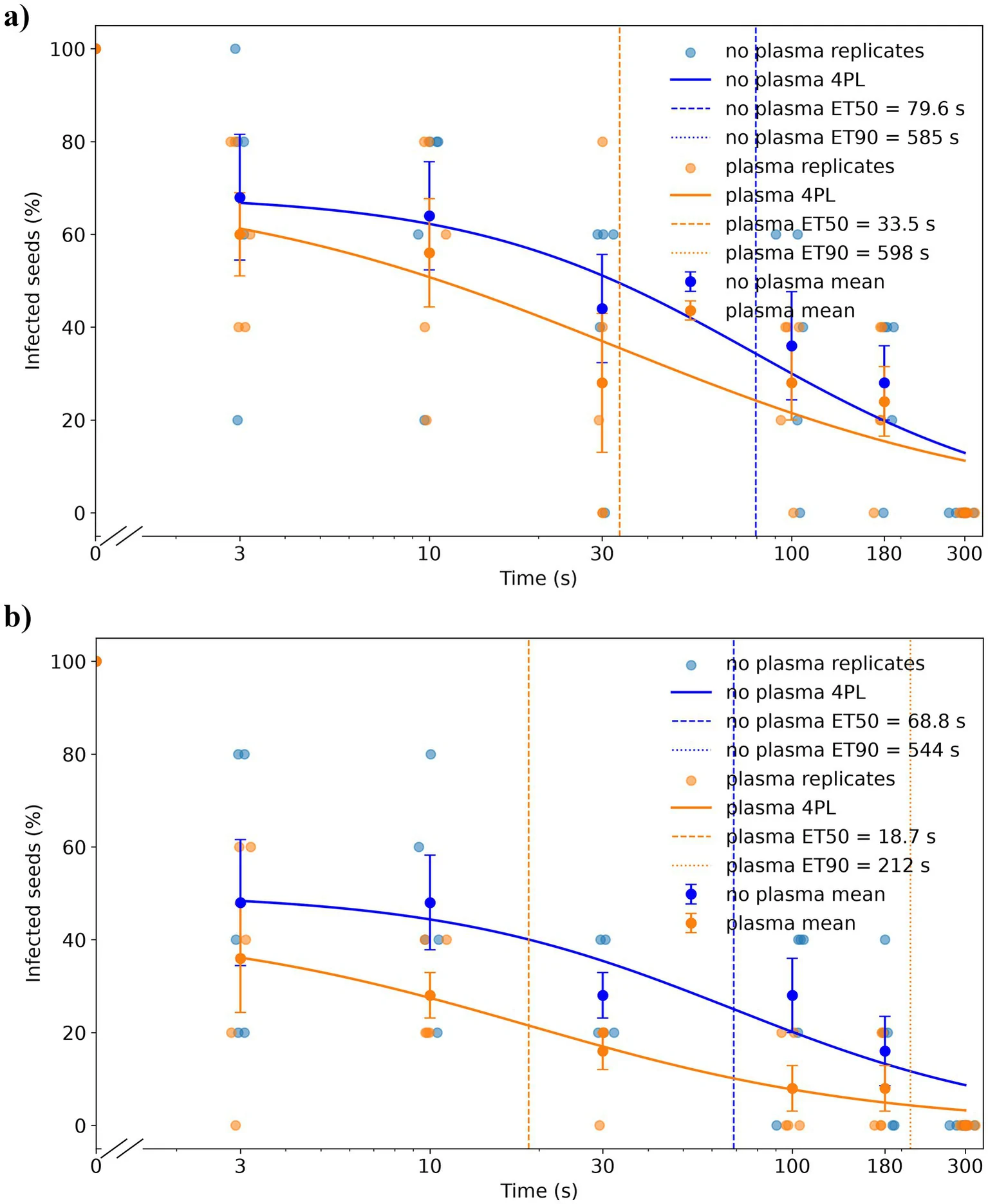
Effect of cold plasma pre-treatment on time-dependent PNA-mediated suppression of fungal infection on maize seeds. Maize seeds inoculated with **(a)** *Fusarium fujikuroi* or **(b)** *Aspergillus flavus* were treated with 100 mM peroxynitric acid (PNA) for increasing exposure times, either without plasma pre-treatment (blue) or after plasma pre-treatment (orange). Individual points represent replicate measurements (*n* = 5), solid symbols show means ± SE, and solid lines represent fitted four-parameter logistic (4PL) models. Vertical dashed and dotted lines indicate model-derived ET_50_ and ET_90_ values, respectively. ET_90_ estimates exceeding the maximum experimentally tested exposure time of 300 s represent model-based extrapolations rather than directly observed treatment thresholds. The x-axis is logarithmic; 0 s controls are displayed at offset positions for visualization only and were not included in model fitting. Both panels are shown on the same x-axis scale to facilitate comparison between species. Detailed statistical outputs are provided in Supplementary Tables S10–S15.

The fitted models showed a numerical shift toward shorter early inactivation times after plasma pre-treatment. ET_50_ decreased from 79.6 s (95% bootstrap CI: 11.5–189.3 s) without plasma to 33.5 s (95% bootstrap CI: 5.41–162.7 s) with plasma. In contrast, ET_90_ estimates were 585 s (95% bootstrap CI: 38.03–1252.0 s) and 598 s (95% bootstrap CI: 11.9–1,153 s), respectively, and both were extrapolated beyond the experimental range. Planned plasma-versus-no-plasma comparisons at individual exposure times were not statistically significant after adjustment for overdispersion. Accordingly, the fitted shifts represent numerical trends rather than statistically confirmed plasma effects. Detailed analyses are provided in Supplementary Tables S10–S12.

For *A. flavus* (Figure 4b), mean infection without plasma was 48, 48, 28, 28, and 16% after 3, 10, 30, 100, and 180 s, respectively, with complete suppression at 300 s. After plasma pre-treatment, mean infection was 36, 28, 16, 8, and 8% at the corresponding exposure times, with complete suppression at 300 s. The plasma-pre-treated group therefore showed lower mean infection at each non-zero exposure time, while the final outcome at 300 s was unchanged.

The fitted ET50 decreased numerically from 68.8 s (95% bootstrap CI: 4.69–189.4 s) without plasma to 18.7 s (95% bootstrap CI: 1.50–94.9 s) after plasma pre-treatment. The fitted ET90 point estimate also decreased numerically, from 544.3 s (95% bootstrap CI: 32.86–830.3 s) to 211.6 s (95% bootstrap CI: 23.6–589.4 s). The ET90 estimate without plasma exceeded the maximum experimentally tested exposure time of 300 s and therefore represents a model-based extrapolation. In contrast, the ET90 point estimate after plasma pre-treatment fell within the tested exposure range, although its 95% bootstrap confidence interval extended beyond 300 s. Planned plasma-versus-no-plasma comparisons at individual exposure times were not statistically significant after adjustment for overdispersion and Holm correction for multiple comparisons. Accordingly, the plasma-associated differences should be interpreted as numerical trends in the fitted response kinetics rather than as statistically confirmed treatment effects. Detailed analyses are provided in Supplementary Tables S13–S15. Taken together, plasma pre-treatment was associated with lower observed infection and shorter model-derived ET_50_ values, particularly for *A. flavus*. However, the adjusted statistical comparisons did not confirm a significant plasma effect, and the confidence intervals around the model-derived exposure times were wide and overlapping. The results therefore support, at most, a possible acceleration of early PNA-mediated inactivation under the tested conditions rather than a demonstrated improvement in final disinfection efficacy.

### 3.3 Germination rate

Germination assays (Figure 5) showed that neither PNA treatment nor plasma exposure caused a statistically significant reduction in seed germination, indicating that the tested treatments did not measurably compromise seed viability under the applied conditions.

**Figure 5:**
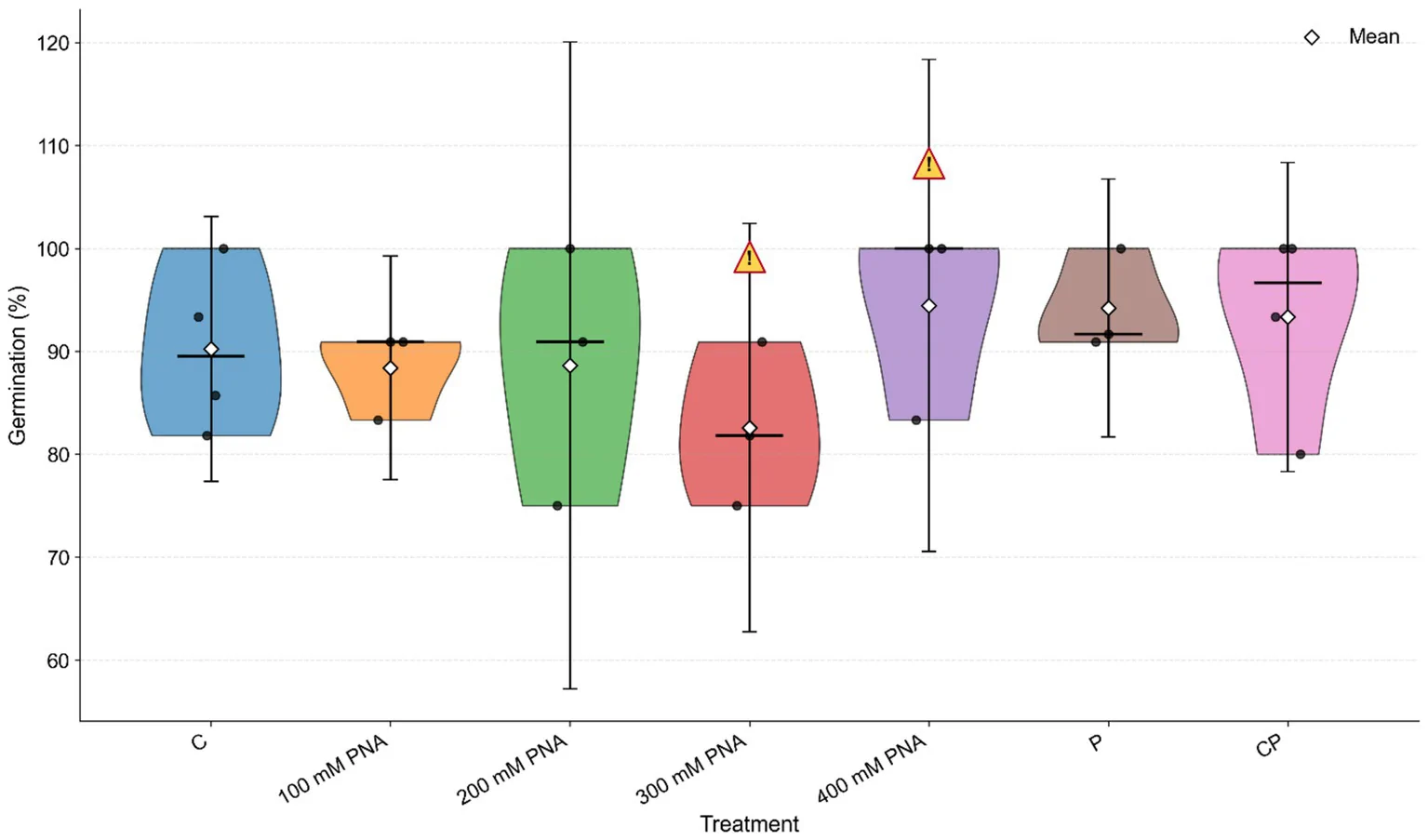
Germination percentage of maize seeds after treatment with PNA, hydrogen peroxide, and cold plasma. Violin plots show the distribution of germination (%) across treatments: control (C), 100–400 mM peroxynitric acid (PNA), hydrogen peroxide (P), and cold plasma (CP). Black points indicate individual replicates, horizontal lines denote medians, and white diamonds represent means with 95% confidence intervals. Warning symbols (⚠) indicate differences exceeding the predefined smallest effect size of interest (SESOI; ±4.09 percentage points). Detailed statistical analyses are provided in Supplementary Tables S16–S19.

Germination remained high across all treatments, with mean values ranging from 82.6% in the 300 mM PNA treatment to 94.4% in the 400 mM PNA treatment (Figure 5). The control (C), hydrogen peroxide-treated seeds (P), and cold plasma-treated seeds (CP) showed consistently high germination, with mean values of 90.2, 94.2, and 93.3%, respectively.

The Kruskal–Wallis test showed no statistically significant overall effect of treatment on germination (H = 5.16, *p* = 0.523). Pairwise comparisons with Holm correction were also not significant. The 300 mM PNA group showed lower mean germination than several comparator groups, with effect sizes exceeding the predefined SESOI in some comparisons; however, these differences were not statistically confirmed and the sample size was limited (*n* = 3–4 per group). Thus, the data provide no statistically supported evidence of reduced germination under the tested conditions, but suggest a possible treatment-specific reduction in germination at 300 mM PNA, warranting further investigation (Supplementary Tables S16–S19).

## 4 Discussion

The present study demonstrates the concentration- and exposure-time-dependent antifungal activity of PNA against *A. flavus* and *F. fujikuroi* on artificially inoculated maize seeds. The concentration–response experiment showed a clear reduction in *F. fujikuroi* infection with increasing PNA concentration, with an estimated IC_50_ of 50.1 mM (95% bootstrap CI: 27.35–94.87 mM) and IC_90_ of 119 mM (95% bootstrap CI: 30.61–209.1 mM). At 180 s, 100 mM PNA produced strong suppression, while 400 mM completely suppressed detectable infection under the experimental conditions. These results extend the previously reported antimicrobial activity of PNA beyond bacterial systems to two filamentous fungal species, although the present data do not establish efficacy against seed-borne fungi generally.

The observed activity is consistent with the known oxidative and nitrative reactivity of PNA and with previous studies showing rapid microbial inactivation by PNA (Yokoyama et al., 2020; Yokoyama et al., 2021). PNA-derived reactive species can potentially damage multiple cellular targets, including membrane components, proteins, and nucleic acids. However, no direct measurements of oxidative damage, intracellular targets, or fungal stress responses were performed in the present study. Therefore, these mechanisms should be regarded as plausible explanations rather than experimentally demonstrated pathways in *A. flavus* and *F. fujikuroi*.

A clear species-dependent difference was observed at 50 mM PNA. *A. flavus* showed faster inactivation than *F. fujikuroi*, with model-derived ET_50_ values of 42.3 s and 95.1 s, respectively. The ET_90_ estimate for *F. fujikuroi* was 389.6 s and extended beyond the experimental range, whereas the estimate for *A. flavus* was 61.1 s. The confidence intervals indicate uncertainty in these model-derived quantities, particularly for ET_90_. The different response shapes, including the steeper fitted transition for *A. flavus*, are consistent with species-specific susceptibility to oxidative stress.

Differences in cell-wall and spore-surface properties, permeability, antioxidant defences, and oxidative-stress response pathways could contribute to this species-specific susceptibility, as suggested for fungal responses to reactive oxygen and nitrogen species and plasma treatments (Hou et al., 2018; Song et al., 2022; Mravlje et al., 2023). However, these mechanisms were not measured here and should therefore be tested experimentally in future work. The present findings support a species-specific interpretation rather than a generalized ranking of fungal susceptibility.

The plasma experiments showed lower mean infection and shorter model-derived ET_50_ values after pre-treatment for both species, with the numerical shift more pronounced for *A. flavus*. However, the planned plasma-versus-no-plasma comparisons were not statistically significant after adjustment for overdispersion. The confidence intervals around the ET estimates were also wide, indicating substantial uncertainty. Therefore, the present study does not provide sufficient statistical evidence to conclude that plasma pre-treatment enhanced PNA-mediated fungal inactivation. The observed shifts are better interpreted as numerical trends suggesting that plasma may influence the early kinetics of PNA treatment under these conditions.

One possible explanation is that plasma altered the seed surface or the local chemical environment in a manner that affected subsequent interaction with PNA. Plasma treatment is known to modify surface chemistry and wettability in some materials and seed systems (Recek et al., 2021; Vesel et al., 2025). However, contact angle, surface chemistry, morphology, or penetration measurements were not performed in this study. Consequently, increased hydrophilicity, improved wetting, enhanced oxidant penetration, or altered local RONS chemistry remain hypotheses and should not be presented as established mechanisms. The very short plasma exposure used here (approximately 0.4 s in total) may also have limited the magnitude of any surface modification. Future experiments combining plasma treatment with contact-angle measurements, SEM/AFM, XPS or FTIR, and chemical analysis of PNA/RONS at the seed surface would be needed to test these explanations directly.

Neither PNA nor plasma treatment produced a statistically significant overall reduction in seed germination. This finding is relevant to the practical use of the treatment because antimicrobial efficacy must be balanced against seed viability. Nevertheless, the lower mean germination observed at 300 mM PNA and the limited number of germination replicates indicate that the absence of statistical significance should not be interpreted as proof of complete physiological safety. Larger germination experiments and additional measures of seed vigor and early seedling development would provide a stronger assessment of treatment compatibility.

The study has several limitations that constrain interpretation and practical extrapolation. First, experiments were performed with artificially inoculated and sterilized maize seeds under controlled laboratory conditions. Naturally contaminated commercial seed lots may differ in fungal load, spatial distribution, background microbiota, organic matter, seed-surface properties, and moisture, all of which could affect PNA demand and treatment efficacy. Second, only *A. flavus* and *F. fujikuroi* were examined; the findings should therefore not be generalized to seed-borne fungi as a group. Third, mechanistic measurements were not included, so the molecular basis of PNA-mediated fungal inactivation and any contribution of plasma-induced surface changes remain unresolved. Fourth, seed quality assessment was limited primarily to germination, and longer-term seed vigor, storage stability, and seedling performance were not evaluated. Finally, fungal viability was measured, but mycotoxin concentrations were not determined; consequently, the study cannot establish whether fungal inactivation would translate into reduced aflatoxin or fumonisin contamination.

From an implementation perspective, PNA has several potentially attractive characteristics as an oxidative disinfectant, including high reactivity and relatively rapid decomposition, but these same properties may complicate storage, transport, and process standardization. The treatment would therefore likely require on-site or near-site preparation, controlled concentration, exposure time, temperature, pH, and monitoring of reactive-species chemistry. Scale-up will also require assessment of PNA generation rate, reagent consumption, worker safety, reactor or soaking-system design, treatment uniformity, wastewater or residual management, and compatibility with existing seed-treatment equipment. Regulatory approval and demonstration of residue safety would be required before agricultural deployment. These practical aspects were not addressed experimentally in the present study and should be investigated alongside efficacy and mycotoxin reduction in future work.

Compared with other oxidative approaches, PNA offers the potential advantage of combining strong oxidizing/nitrating reactivity with relatively short persistence. Hydrogen peroxide, ozone, and cold plasma have also been investigated for seed or agricultural-material decontamination, but their performance depends strongly on dose, exposure time, seed properties, and treatment configuration. Cold plasma has the additional advantage of being non-thermal and capable of generating multiple RONS, although treatment reproducibility and scale-up can be affected by reactor configuration and process conditions (Mravlje et al., 2021; Cavalcanti et al., 2023). Ozone is another residue-minimizing oxidant that has been investigated for microbial and mycotoxin decontamination, but its effectiveness and potential effects on seed quality are also dose- and matrix-dependent. Importantly, fungal inactivation does not necessarily imply mycotoxin removal; therefore, future comparisons should evaluate both viable fungal populations and toxin concentrations.

Overall, the present results support PNA as a promising candidate for further investigation rather than as a validated replacement for conventional fungicides. Validation with naturally contaminated commercial seed lots, mechanistic characterization of fungal inactivation and plasma–PNA interactions, assessment of mycotoxin reduction, and evaluation of process economics, stability, safety, and scale-up are necessary before practical application can be established.

## 5 Conclusion

Peroxynitric acid (PNA) demonstrated rapid and highly effective antifungal activity against the seed-borne pathogens *A. flavus* and *F. fujikuroi* on maize seeds. Antifungal efficacy was strongly dependent on both concentration and exposure time, with 100 mM PNA achieving strong suppression after prolonged treatment. The two fungal species exhibited markedly different inactivation kinetics, with *A. flavus* being substantially more susceptible to oxidative treatment than *F. fujikuroi*. Non-equilibrium plasma pre-treatment was associated with numerically shorter model-derived exposure times and faster early inactivation kinetics; particularly for *A. flavus*; however, plasma-versus-no-plasma differences were not statistically significant after adjustment for overdispersion. These findings therefore indicate a numerical trend toward faster inactivation following plasma pre-treatment rather than a confirmed enhancement of final PNA-mediated disinfection. The estimated effects and their confidence intervals should consequently be considered when interpreting the magnitude and precision of the observed plasma-associated differences.

Importantly, neither PNA nor plasma treatment caused a statistically significant reduction in seed germination under the tested conditions, supporting the potential applicability of this approach for seed disinfection. However, the present study was conducted using artificially inoculated and sterilized maize seeds under controlled laboratory conditions. Therefore, the effectiveness of PNA-based treatment against naturally contaminated commercial seed lots, where fungal loads, microbial communities, seed-surface characteristics, and organic matter may differ substantially, remains to be established. Further research should evaluate the treatment using naturally contaminated seeds and commercially relevant conditions, investigate the underlying mechanisms governing PNA-mediated fungal inactivation and the potential contribution of plasma-induced surface and chemical changes, and determine whether treatment can reduce mycotoxin contamination in addition to viable fungal populations. Such studies will be important for assessing the robustness, safety, and practical applicability of PNA-based seed disinfection.

Overall, the present findings demonstrate the strong concentration- and time-dependent antifungal activity of PNA against *A. flavus* and *F. fujikuroi* and provide evidence of species-specific differences in oxidative inactivation responses. While plasma pre-treatment showed numerical trends toward faster early inactivation, the absence of statistically significant plasma-versus-no-plasma differences means that its contribution to PNA-mediated fungal disinfection remains to be confirmed. PNA therefore represents a promising candidate for further investigation as an alternative approach to conventional chemical seed treatments, particularly in combination with mechanistic studies and validation using naturally contaminated seeds and mycotoxin endpoints.

## Data Availability

The datasets presented in this study can be found in online repositories. The names of the repository/repositories and accession number(s) can be found in the article/Supplementary material. Open public repository Zenodo: https://doi.org/10.5281/zenodo.21103290.

## References

[ref1] Agresti A. (2013). Categorical data analysis. Hoboken, NJ, USA: John Wiley & Sons.

[ref2] Akpor O. B. Ajinde A. O. Dayo-Olagbende O. G. (2024). Non-thermodynamic equilibrium plasma, an oxidation process for environmental protection: principles, mechanisms, and prospects 2024 International Conference on Science, Engineering and Business for Driving Sustainable Development Goals (SEB4SDG). 1–17

[ref3] Anand G. Rajeshkumar K. C. (2022). “Challenges and threats posed by plant pathogenic fungi on agricultural productivity and economy,” in Fungal Diversity, Ecology and Control Management. Eds. Rajpal V.R. Singh I. Navi S.S. (Singapore: Springer), 483–493.

[ref4] Awuchi C. G. Ondari E. N. Ogbonna C. U. Upadhyay A. K. Baran K. Okpala C. O. R. . (2021). Mycotoxins affecting animals, foods, humans, and plants: types, occurrence, toxicities, action mechanisms, prevention, and detoxification strategies—a revisit. Foods 10:1279. doi: 10.3390/foods10061279, 34205122 PMC8228748

[ref5] Ayesha M. S. Suryanarayanan T. S. Nataraja K. N. Prasad S. R. Shaanker R. U. (2021). Seed treatment with systemic fungicides: time for review. Front. Plant Sci. 12:654512. doi: 10.3389/fpls.2021.654512 PMC8365024 34408757

[ref6] Bilea F. Garcia-Vaquero M. Magureanu M. Mihaila I. Mildaziene V. Mozetic M. . (2024). Non-thermal plasma as environmentally-friendly technology for agriculture: a review and roadmap. Crit. Rev. Plant Sci. 43, 428–486. doi: 10.1080/07352689.2024.2410145

[ref7] Cavalcanti R. N. Pimentel T. C. Esmerino E. A. de Freitas M. Q. Verruck S. Silva M. C. . (2023). “Cold plasma,” in Novel Technologies in Food Science. Eds. Chhikara N. Panghal A. Chaudhary G. (Beverly, MA, USA: Scrivener Publishing LLC), 109–169.

[ref8] Christensen C. M. (1957). Deterioration of stored grains by fungi. Bot. Rev., 23, 108–134.

[ref9] Cohen J. (2013). Statistical power Analysis for the Behavioral Sciences. New York, NY: Routledge.

[ref10] Fantz U. Briefi S. Rauner D. Wuenderlich D. (2016). Quantification of the VUV radiation in low pressure hydrogen and nitrogen plasmas. Plasma Sources Sci. Technol. 25:045006. doi: 10.1088/0963-0252/25/4/045006

[ref11] Fisher M. C. Gow N. A. Gurr S. J. (2016). Tackling Emerging Fungal Threats to animal Health, Food Security and Ecosystem Resilience. London, UK: The Royal Society. 10.1098/rstb.2016.0332 PMC5095550 28080997

[ref12] Fung F. Clark R. F. (2004). Health effects of mycotoxins: a toxicological overview. J. Toxicol. Clin. Toxicol. 42, 217–234. doi: 10.1081/CLT-120030947, 15214629

[ref13] Gupta A. Kumar R. (2020). “Management of seed-borne diseases: an integrated approach,” in Seed-borne Diseases of Agricultural Crops: Detection, Diagnosis & Management. Eds. Kumar R. Gupta A. (Singapore: Springer), 717–745.

[ref14] Harikrishna S. Anil P. P. Shams R. Dash K. K. (2023). Cold plasma as an emerging nonthermal technology for food processing: a comprehensive review. J. Agric. Food Res. 14:100747. doi: 10.1016/j.jafr.2023.100747

[ref15] Hedges L. V. (1981). Distribution theory for glass's estimator of effect size and related estimators. J. Educ. Stat. 6, 107–128. doi: 10.3102/10769986006002107

[ref16] Holländer A. Behnisch J. (1998). Vacuum-ultraviolet photolysis of polymers. Surf. Coat. Technol. 98, 855–858. doi: 10.1016/S0257-8972(97)00128-X

[ref17] Holm S. (1979). A simple sequentially rejective multiple test procedure. Scand. J. Stat., 6, 65–70. Available online at: http://www.jstor.org/stable/4615733 (Accessed September 16, 2026).

[ref18] Hou Y. P. Qu X. P. Mao X. W. Kuang J. Duan Y. B. Song X. S. . (2018). Resistance mechanism of *Fusarium fujikuroi* to phenamacril in the field. Pest Manag. Sci. 74, 607–616. doi: 10.1002/ps.4742 28960890

[ref19] Ikawa S. Tani A. Nakashima Y. Kitano K. (2016). Physicochemical properties of bactericidal plasma-treated water. J. Phys. D. Appl. Phys. 49:425401. doi: 10.1088/0022-3727/49/42/425401

[ref20] Iwaki T. Ohshima T. Tasaki T. Momoi Y. Ikawa S. Kitano K. . (2020). High microbicidal effect of peroxynitric acid on biofilm-infected dentin in a root carious tooth model and verification of tissue safety. J. Oral Biosci. 62, 189–194. doi: 10.1016/j.job.2020.03.002 32272186

[ref21] Klich M. A. (2007). *Aspergillus flavus*: the major producer of aflatoxin. Mol. Plant Pathol. 8, 713–722. doi: 10.1111/j.1364-3703.2007.00436.x 20507532

[ref22] Lakens D. (2017). Equivalence tests: a practical primer for t tests, correlations, and meta-analyses. Soc. Psychol. Personal. Sci. 8, 355–362. doi: 10.1177/1948550617697177, 28736600 PMC5502906

[ref23] Lamichhane J. R. You M. P. Laudinot V. Barbetti M. J. Aubertot J.-N. (2020). Revisiting sustainability of fungicide seed treatments for field crops. Plant Dis. 104, 610–623. doi: 10.1094/PDIS-06-19-1157-FE 31944170

[ref24] Løgager T. Sehested K. (1993). Formation and decay of peroxynitrous acid: a pulse radiolysis study. J. Phys. Chem. 97, 6664–6669.

[ref25] Mancini V. Romanazzi G. (2014). Seed treatments to control seedborne fungal pathogens of vegetable crops. Pest Manag. Sci. 70, 860–868. doi: 10.1002/ps.3693 24293285

[ref26] Mesterhazy A. (2024). Food safety aspects of breeding maize to multi-resistance against the major (*Fusarium graminearum*, *F. verticillioides*, *Aspergillus flavus*) and minor toxigenic fungi (*Fusarium* spp.) as well as to toxin accumulation, trends, and solutions—a review. J. Fungi 10:40. doi: 10.3390/jof10010040, 38248949 PMC10817526

[ref27] Mitchell N. J. Bowers E. Hurburgh C. Wu F. (2016). Potential economic losses to the US corn industry from aflatoxin contamination. Food Addit. Contam. Part A Chem. Anal. Control Expo. Risk Assess. 33, 540–550. doi: 10.1080/19440049.2016.1138545, 26807606 PMC4815912

[ref28] Moreau M. Orange N. Feuilloley M. (2008). Non-thermal plasma technologies: new tools for bio-decontamination. Biotechnol. Adv. 26, 610–617. doi: 10.1016/j.biotechadv.2008.08.001 18775485

[ref29] Mravlje J. Kobal T. Regvar M. Staric P. Zaplotnik R. Mozetic M. . (2023). The sensitivity of fungi colonising buckwheat grains to cold plasma is species specific. J. Fungi 9:609. doi: 10.3390/jof9060609 PMC10301608 37367545

[ref30] Mravlje J. Regvar M. Staric P. Mozetic M. Vogel-Mikus K. (2021). Cold plasma affects germination and fungal community structure of buckwheat seeds. Plants 10:851. doi: 10.3390/plants10050851 PMC8145130 33922511

[ref31] Munkvold G. P. (2016). “Fusarium species and their associated mycotoxins,” in Mycotoxigenic Fungi: Methods and Protocols. Eds. Moretti A. Susca A. (New York, NY: Humana Press), 51–106. 10.1007/978-1-4939-6707-0_4 27924531

[ref32] Muskett A. Malone J. (1941). The Ulster Method for the Examination of Flax Seed for the Presence of Seed-borne Parasites. Ed. Brierley W.B. (Chichester, UK: John Wiley & Sons, Ltd.).

[ref33] Nakashima Y. Ikawa S. Tani A. Kitano K. (2016). Ion-exchange chromatographic analysis of peroxynitric acid. J. Chromatogr. A 1431, 89–93. doi: 10.1016/j.chroma.2015.12.054, 26748867

[ref34] Placinta C. D'Mello J. F. Macdonald A. (1999). A review of worldwide contamination of cereal grains and animal feed with *fusarium* mycotoxins. Anim. Feed Sci. Technol. 78, 21–37. doi: 10.1016/s0377-8401(98)00278-8

[ref35] Pogoda A. Hahn V. Weltmann K.-D. Kolb J. F. (2026). Sporicidal and bactericidal efficacy of plasma-treated liquids based on reaction kinetics of peroxynitrous acid. Front. Microbiol. 17:2026. doi: 10.3389/fmicb.2026.1788374 PMC13008925 41883796

[ref36] Primc G. Paul D. Mozetič M. Kovač J. Vesel A. (2021). “Atomic species generation by plasmas,” in Plasma Applications for Material Modification. Eds. Mozetič M. Cvelbar U. Vesel A. (Singapore: Jenny Stanford Publishing), 107–176.

[ref37] Recek N. Vesel A. Zaplotnik R. Paul D. Primc G. Gselman P. . (2021). Hydrophilization of corn seeds by non-equilibrium gaseous plasma. Chem. Biol. Technol. Agric. 8:32. doi: 10.1186/s40538-021-00231-w, 38164791

[ref38] Rezaei F. Vanraes P. Nikiforov A. Morent R. De Geyter N. (2019). Applications of plasma-liquid systems: a review. Materials 12:2751. doi: 10.3390/ma12172751, 31461960 PMC6747786

[ref39] Ritz C. Baty F. Streibig J. C. Gerhard D. (2015). Dose-response analysis using R. PLoS One 10:e0146021. doi: 10.1371/journal.pone.0146021, 26717316 PMC4696819

[ref40] Ritz C. Streibig J. C. (2005). Bioassay analysis using R. J. Stat. Softw. 12, 1–22. doi: 10.18637/jss.v012.i05, 42722280

[ref41] Scholtz V. Vaňková E. Kašparová P. Premanath R. Karunasagar I. Julák J. (2021). Non-thermal plasma treatment of ESKAPE pathogens: a review. Front. Microbiol. 12:737635. doi: 10.3389/fmicb.2021.737635, 34712211 PMC8546340

[ref42] Sharma A. Kumar V. Shahzad B. Tanveer M. Sidhu G. P. S. Handa N. . (2019). Worldwide pesticide usage and its impacts on ecosystem. SN Appl. Sci. 1:1446. doi: 10.1007/s42452-019-1485-1

[ref43] Singh U. B. Chaurasia R. Manzar N. Kashyap A. S. Malviya D. Singh S. . (2020). “Chemical management of seed-borne diseases: achievements and future challenges,” in Seed-Borne Diseases of Agricultural Crops: Detection, Diagnosis & Management. Eds. Kumar R. Gupta A. (Singapore: Springer), 665–682. doi: 10.1007/978-981-32-9046-4_23

[ref44] Singh I. Thakur P. (2023). “Impact of fungi on the world economy and its sustainability: current status and potentials,” in Fungal Resources for Sustainable Economy: Current Status and Future Perspectives. Eds. Singh I. Rajpal V.R. Navi S.S. (Singapore: Springer), 3–37. doi: 10.1007/978-981-19-9103-5_1

[ref45] Song Y. Chen X. Sun J. Bai Y. Jin L. Lin Y. . (2022). *In vitro* determination of sensitivity of *Fusarium fujikuroi* to fungicide azoxystrobin and investigation of resistance mechanism. J. Agric. Food Chem. 70, 9760–9768. doi: 10.1021/acs.jafc.2c02663 35901518

[ref46] Vesel A. Macovei A. Balestrazzi A. Tondepu S. A. G. Duenas C. Jr. Locato V. . (2025). Hydrophilization of rice seeds by plasma treatments–super-hydrophilic surface finish and hydrophobic recovery. Appl. Surf. Sci. 691:162674. doi: 10.1016/j.apsusc.2025.162674

[ref47] Watson S. Abdelmalek F. Larouche É. Généreux M. Monette J. Côté C. . (2025). Electrifying greenhouse agriculture: cold atmospheric pressure plasma technology for *Pythium ultimum* control. Environ. Technol. Innov. 39:104222. doi: 10.1016/j.eti.2025.104222

[ref48] Xu Y. Bassi A. (2025). Non-thermal plasma decontamination of microbes: a state of the art. Biotechnol. Prog. 41:e3511. doi: 10.1002/btpr.3511, 39462867 PMC12000644

[ref49] Yohannis E. Urugo M. M. Teka T. A. Getachew P. Tola Y. B. Forsido S. F. . (2025). Aflatoxin contamination in Agri-food systems: a comprehensive review of toxicity, food security, economic impacts, and sustainable mitigation across the value chain. Food Sci. Nutr. 13:e71104. doi: 10.1002/fsn3.71104, 41111890 PMC12529245

[ref50] Yokoyama T. Miyazaki S. Akagi H. Ikawa S. Kitano K. (2021). Kinetics of bacterial inactivation by peroxynitric acid in the presence of organic contaminants. Appl. Environ. Microbiol. 87, e01860–e01820. doi: 10.1128/AEM.01860-20, 33127816 PMC7783340

[ref51] Yokoyama T. Miyazaki S. Ikawa S. Nakashima Y. Kitano K. (2020). Kinetics analysis of the reactions between Peroxynitric acid and amino acids. Chem. Res. Toxicol. 33, 1633–1643. doi: 10.1021/acs.chemrestox.9b00408, 32298095

[ref52] Zver M. Dobnik D. Mozetič M. Vesel A. Filipić A. Kogovšek P. . (2024). Antiviral respiratory masks with plasma-functionalized polypropylene textiles for optimal adsorption of antiviral substance. Prog. Org. Coat. 197:108827. doi: 10.1016/j.porgcoat.2024.108827

