# Supplementary material for: Plasma pre-treatment and peroxynitric acid soaking as a novel oxidative seed disinfection strategy against seed-borne fungal pathogens in maize
Source: Front Microbiol. 2026 Sep 28;17:1927228. doi: 10.3389/fmicb.2026.1927228 (PMC13654942; doi:10.3389/fmicb.2026.1927228)
Supplement: Supplementary file 1 [file Supplementary_file_1.docx]

Supplementary Methods – Statistical Analysis Details

The following sections complement the Statistical analysis section of the main manuscript and provide implementation, uncertainty, diagnostic, effect-size, and equivalence details that are not repeated in the main text.

# S1. Supplementary statistical methods

## S1.1 Overdispersion and dispersion-adjusted inference

For the binomial generalized linear models, replicate-level infection proportions were fitted with the number of seeds assessed per replicate used as variance weights. Overdispersion was quantified using the Pearson dispersion parameter:

φ = (Σᵢ rₚ,ᵢ²) / dfᵣₑₛ

where rₚ,ᵢ is the Pearson residual for observation i and dfᵣₑₛ is the residual degrees of freedom. Values of φ > 1 indicate extra-binomial variation. For the overall treatment test, the reduction in model deviance between the null and full models was scaled by the estimated dispersion parameter:

F = [ (D₀ − D₁) / df₁ ] / φ

where D₀ and D₁ are the null- and full-model deviances, respectively, df₁ is the difference in model degrees of freedom, and the denominator degrees of freedom correspond to the residual degrees of freedom of the full model.

For pairwise two-proportion comparisons, the nominal z statistic was adjusted for overdispersion as:

zₐdⱼ = z / √φ

Two-sided p-values were calculated from the adjusted statistic when φ > 1. Holm correction was then applied across the relevant set of pairwise comparisons. Both nominal and dispersion-adjusted results may be retained in the supplementary output tables, but inference in the presence of overdispersion was based on the dispersion-adjusted values.

## S1.2 Effect sizes for infection data

Cohen’s h was used as the primary standardized effect size for differences between infection proportions:

h = 2[sin⁻¹(√p₁) − sin⁻¹(√p₂)]

where p₁ and p₂ are the infection proportions of the two groups being compared (Cohen, 2013). Hedges’ g was additionally calculated from replicate-level responses as an auxiliary standardized effect size. It was obtained from Cohen’s d using the small-sample correction:

g = Jd

J = 1 − 3 / (4df − 1)

df = n₁ + n₂ − 2

where n₁ and n₂ are the numbers of replicate observations in the two groups (Hedges, 1981). Effect-size estimates were interpreted together with, rather than as replacements for, the corresponding inferential tests.

## S1.3 4PL fitting implementation and uncertainty estimation

The four-parameter logistic model defined in the main Statistical analysis section was fitted to the mean response at each positive concentration or exposure-time level using bounded nonlinear least squares.

For the general concentration- and single-treatment time-response workflow, the initial asymptote estimates were the responses at the first and last positive x levels, the initial midpoint was the geometric mean of the positive x values, and the initial Hill slope was 2. The asymptote bounds extended 25% beyond the observed response range, the midpoint was bounded between one-thousandth of the smallest positive x value and 1000 times the largest positive x value, and the Hill slope was constrained to 0.05–30.

For grouped plasma/PNA time-response curve fitting, several starting-value combinations were evaluated for the two asymptotes, midpoint, and Hill slope. Candidate Hill slopes were 0.7, 1, 2, and 4, and both the geometric mean and median of the positive exposure times were evaluated as starting midpoint values. The midpoint was bounded between one-hundredth of the smallest positive time and 20 times the largest observed time, and the Hill slope was bounded between 0.05 and 12. The converged solution with the lowest residual sum of squares was retained.

For grouped fits, residual sum of squares (RSS) and root mean square error (RMSE) were recorded as descriptive measures of agreement between the observed mean responses and fitted curves:

RSS = Σᵢ(yᵢ − ŷᵢ)²

RMSE = √[(1/n) Σᵢ(yᵢ − ŷᵢ)²]

Model assessment was based on numerical convergence, finite and plausible parameter estimates, agreement between the fitted curve and observed mean responses, RSS/RMSE for grouped fits, and the stability of model-derived endpoints under bootstrap resampling. No formal alternative-model selection based on Weibull, Gompertz, AIC, or AICc was performed.

Bootstrap confidence intervals were obtained by resampling replicate responses with replacement within each positive concentration or exposure-time level, recalculating the mean response at each level, and refitting the 4PL model. Only bootstrap fits with finite, positive midpoint and Hill-slope estimates were retained. The 95% confidence interval for each model-derived target was defined by the 2.5th and 97.5th percentiles of the successful bootstrap estimates. A confidence interval was not reported when fewer than 10 successful fits, or fewer than 10% of the requested bootstrap iterations, were obtained. A fixed random seed of 123 was used for reproducibility. The requested number of bootstrap iterations and the number of successful fits were recorded in the corresponding analysis outputs.

## S1.4 Germination effect sizes, biological relevance, and equivalence

In addition to the nonparametric significance tests reported in the main Methods, global effect-size estimates (η² and ε²) and pairwise Hedges’ g values were calculated to describe the magnitude of treatment differences in germination. These effect sizes were treated as descriptive measures because of the small number of replicate dishes per treatment.

Practical biological relevance was evaluated using the coefficient-of-variation-based smallest effect size of interest (SESOI) implemented in the germination analysis. This procedure yielded a prespecified margin of ±4.09 percentage points. Absolute treatment differences exceeding this margin were flagged as potentially biologically relevant even when the corresponding pairwise test was not statistically significant.

Equivalence to the untreated control was evaluated using two one-sided tests (TOST) with equivalence bounds fixed at −4.09 and +4.09 percentage points (Lakens, 2017). Equivalence was concluded only when both one-sided tests supported that the treatment-control difference lay within these bounds. Failure to demonstrate equivalence was interpreted as inconclusive evidence regarding similarity and not as evidence of a treatment effect.

## S1.5 Supplementary statistical outputs

Complete model parameters, bootstrap confidence intervals, pairwise comparisons, dispersion estimates, effect sizes, and germination analyses are reported in Supplementary Tables S1–S19.

## References cited in the Supplementary Methods

Cohen, J. (2013). Statistical Power Analysis for the Behavioral Sciences. Routledge.

Hedges, L. V. (1981). Distribution theory for Glass’s estimator of effect size and related estimators. Journal of Educational Statistics, 6, 107–128.

Holm, S. (1979). A simple sequentially rejective multiple test procedure. Scandinavian Journal of Statistics, 6, 65–70.

Lakens, D. (2017). Equivalence tests: A practical primer for t tests, correlations, and meta-analyses. Social Psychological and Personality Science, 8, 355–362.
