# Supplementary material for: Plasma pre-treatment and peroxynitric acid soaking as a novel oxidative seed disinfection strategy against seed-borne fungal pathogens in maize
Source: Front Microbiol. 2026 Sep 28;17:1927228. doi: 10.3389/fmicb.2026.1927228 (PMC13654942; doi:10.3389/fmicb.2026.1927228)
Supplement: Supplementary file 2 [file Supplementary_file_2.docx]

**Supplementary Tables**

Tables summarize the statistical analyses supporting the concentration-response, time-response, plasma pre-treatment and germination experiments. Binomial seed-infection analyses are based on infected/non-infected seed counts; germination analyses are based on replicate-level germination percentages.

# Figure 2: concentration-response analysis

**Supplementary Table S1. Descriptive statistics of concentration-dependent reduction of Fusarium fujikuroi infection in artificially inoculated maize seeds after 180 s exposure to PNA.**

Wilson confidence intervals are based on pooled infected/non-infected seed counts within each concentration. Each replicate consisted of 100 seeds.

| **PNA concentration** | **Replicates (n)** | **Mean infected seeds (%)** | **SD** | **SE** | **Median** | **Min** | **Max** | **Infected seeds (count)** | **Total seeds assessed** | **Infected seeds (%) from counts** | **Wilson 95% CI** |
| --- | --- | --- | --- | --- | --- | --- | --- | --- | --- | --- | --- |
| 0 mM | 5 | 100 | 0 | 0 | 100 | 100 | 100 | 500 | 500 | 100 | 99.24 to 100.0 |
| 1 mM | 5 | 100 | 0 | 0 | 100 | 100 | 100 | 500 | 500 | 100 | 99.24 to 100.0 |
| 10 mM | 5 | 100 | 0 | 0 | 100 | 100 | 100 | 500 | 500 | 100 | 99.24 to 100.0 |
| 100 mM | 5 | 16 | 16.73 | 7.48 | 20 | 0 | 40 | 80 | 500 | 16 | 13.05 to 19.47 |
| 200 mM | 5 | 10 | 22.36 | 10 | 0 | 0 | 50 | 50 | 500 | 10 | 7.67 to 12.94 |
| 300 mM | 6 | 3.33 | 8.16 | 3.33 | 0 | 0 | 20 | 20 | 600 | 3.33 | 2.17 to 5.09 |
| 400 mM | 5 | 0 | 0 | 0 | 0 | 0 | 0 | 0 | 500 | 0 | 0.00 to 0.76 |

**Supplementary Table S2. Overall test, overdispersion and 4PL concentration-response model parameters for Figure 2.**

IC values are model-derived concentrations associated with the stated percentage reduction in infection. Confidence intervals were estimated by bootstrap percentile intervals.

| **Parameter** | **Value** |
| --- | --- |
| Analysis type | binomial_count |
| Reported metric | IC |
| Target levels (%) | 10, 50, 90 |
| Primary model | fallback chi-square test: infected/noninfected counts by group |
| Overall test statistic | 3070.2 |
| Overall test df | 6.0000 |
| Overall test p-value | <0.0001 |
| Overdispersion (Pearson chi-square/df) | 14.10 |
| IC10 (mM) | 20.99 |
| IC10 95% bootstrap CI, lower (mM) | 16.50 |
| IC10 95% bootstrap CI, upper (mM) | 85.94 |
| IC50 (mM) | 50.06 |
| IC50 95% bootstrap CI, lower (mM) | 27.35 |
| IC50 95% bootstrap CI, upper (mM) | 94.87 |
| IC50 log-scale CI status | within measured positive range |
| IC90 (mM) | 119.4 |
| IC90 95% bootstrap CI, lower (mM) | 30.61 |
| IC90 95% bootstrap CI, upper (mM) | 209.1 |
| Successful bootstrap fits | 200.0 |
| Bootstrap iterations | 200.0 |
| 4PL low-dose asymptote (%) | 100.00 |
| 4PL high-dose asymptote (%) | 2.6475 |
| 4PL Hill slope | 2.5280 |
| 4PL RMSE on group means | 2.3860 |
| 4PL reliability | interpret with caution |
| 4PL warnings | Low-dose asymptote is close to a response bound. |
| 4PL fitting note | 4PL fitted on positive x levels only using multiple starting values and maxfev=200000. IC50 CI uses a log-scale approximation. Response asymptotes constrained to 0...100. |
| Overall test note | Because a quasibinomial GLM was not fitted in the final workflow, the overall concentration effect was evaluated using a fallback chi-square test on infected/non-infected counts, with overdispersion reported separately |

**Supplementary Table S3. Pairwise concentration comparisons and effect sizes for Figure 2.**

Pairwise comparisons are based on two-proportion z-tests. The Holm p-value adjusted for overdispersion is the preferred p-value because overdispersion was high. Cohen's h is the recommended effect size for proportions; Hedges' g is included as an auxiliary standardized mean-difference metric and may be undefined when variance is zero.

| **Comparison** | **Difference (percentage points)** | **Holm p-value adjusted for overdispersion** | **Cohen's h** | **Hedges' g** | **Note** |
| --- | --- | --- | --- | --- | --- |
| 0 mM vs 1 mM | 0 | 1.000 | 0 | 0.000 |  |
| 0 mM vs 10 mM | 0 | 1.000 | 0 | 0.000 |  |
| 0 mM vs 100 mM | 84 | <0.0001 | 2.319 | 6.412 |  |
| 0 mM vs 200 mM | 90 | <0.0001 | 2.498 | 5.141 |  |
| 0 mM vs 300 mM | 96.67 | <0.0001 | 2.774 | 14.52 |  |
| 0 mM vs 400 mM | 100 | <0.0001 | 3.142 | not finite | effect size not finite because one or both groups have zero variance |
| 1 mM vs 10 mM | 0 | 1.000 | 0 | 0.000 |  |
| 1 mM vs 100 mM | 84 | <0.0001 | 2.319 | 6.412 |  |
| 1 mM vs 200 mM | 90 | <0.0001 | 2.498 | 5.141 |  |
| 1 mM vs 300 mM | 96.67 | <0.0001 | 2.774 | 14.52 |  |
| 1 mM vs 400 mM | 100 | <0.0001 | 3.142 | not finite | effect size not finite because one or both groups have zero variance |
| 10 mM vs 100 mM | 84 | <0.0001 | 2.319 | 6.412 |  |
| 10 mM vs 200 mM | 90 | <0.0001 | 2.498 | 5.141 |  |
| 10 mM vs 300 mM | 96.67 | <0.0001 | 2.774 | 14.52 |  |
| 10 mM vs 400 mM | 100 | <0.0001 | 3.142 | not finite | effect size not finite because one or both groups have zero variance |
| 100 mM vs 200 mM | 6 | 1.000 | 0.18 | 0.274 |  |
| 100 mM vs 300 mM | 12.67 | 0.421 | 0.456 | 0.911 |  |
| 100 mM vs 400 mM | 16 | 0.117 | 0.823 | 1.221 |  |
| 200 mM vs 300 mM | 6.67 | 1.000 | 0.276 | 0.379 |  |
| 200 mM vs 400 mM | 10 | 0.421 | 0.644 | 0.571 |  |
| 300 mM vs 400 mM | 3.33 | 1.000 | 0.367 | 0.501 |  |

Abbreviations: IC10, IC50 and IC90, effective concentrations associated with 10%, 50% and 90% reduction in infection, respectively; SE, standard error; SD, standard deviation; 4PL, four-parameter logistic model.

# Figure 3a: time-response analysis against Fusarium fujikuroi

**Supplementary Table S4. Descriptive statistics of time-dependent antifungal efficacy of 50 mM PNA against Fusarium fujikuroi inoculated onto maize seeds.**

Wilson confidence intervals are based on pooled infected/non-infected seed counts within each exposure time. Each replicate consisted of 100 seeds.

| **PNA exposure time** | **Replicates (n)** | **Mean infected seeds (%)** | **SD** | **SE** | **Median** | **Min** | **Max** | **Infected seeds (count)** | **Total seeds assessed** | **Infected seeds (%) from counts** | **Wilson 95% CI** |
| --- | --- | --- | --- | --- | --- | --- | --- | --- | --- | --- | --- |
| 0 s | 5 | 100 | 0 | 0 | 100 | 100 | 100 | 500 | 500 | 100 | 99.24 to 100.0 |
| 3 s | 5 | 96 | 8.94 | 4 | 100 | 80 | 100 | 480 | 500 | 96 | 93.90 to 97.40 |
| 10 s | 5 | 92 | 17.89 | 8 | 100 | 60 | 100 | 460 | 500 | 92 | 89.29 to 94.07 |
| 30 s | 5 | 72 | 10.95 | 4.9 | 80 | 60 | 80 | 360 | 500 | 72 | 67.91 to 75.76 |
| 100 s | 5 | 56 | 16.73 | 7.48 | 60 | 40 | 80 | 280 | 500 | 56 | 51.62 to 60.29 |
| 180 s | 5 | 16 | 16.73 | 7.48 | 20 | 0 | 40 | 80 | 500 | 16 | 13.05 to 19.47 |

**Supplementary Table S5. Overall test, overdispersion and 4PL time-response model parameters for Figure 3a.**

ET values are exposure times required to reduce infection by the stated percentage. Confidence intervals were estimated by bootstrap percentile intervals. ET90 exceeds the experimentally tested positive time range and should therefore be interpreted as an extrapolated model descriptor.

| **Parameter** | **Value** |
| --- | --- |
| Analysis type | binomial_count |
| Reported metric | ET |
| Target levels (%) | 10, 50, 90 |
| Primary model | fallback chi-square test: infected/noninfected counts by group |
| Overall test statistic | 1277.8 |
| Overall test df | 5.0000 |
| Overall test p-value | <0.0001 |
| Overdispersion (Pearson chi-square/df) | 17.08 |
| ET10 (s) | 23.22 |
| ET10 95% bootstrap CI, lower (s) | 8.8119 |
| ET10 95% bootstrap CI, upper (s) | 81.30 |
| ET50 (s) | 95.11 |
| ET50 95% bootstrap CI, lower (s) | 62.88 |
| ET50 95% bootstrap CI, upper (s) | 135.2 |
| ET50 log-scale CI status | unstable; prefer bootstrap CI |
| ET90 (s) | 389.6 |
| ET90 95% bootstrap CI, lower (s) | 158.9 |
| ET90 95% bootstrap CI, upper (s) | 767.6 |
| Successful bootstrap fits | 200.0 |
| Bootstrap iterations | 200.0 |
| 4PL low-time asymptote (%) | 93.45 |
| 4PL high-time asymptote (%) | 0.0000 |
| 4PL Hill slope | 1.5583 |
| 4PL RMSE on group means | 7.5608 |
| 4PL reliability | extrapolated; interpret with caution |
| 4PL warnings | Only 5 positive x levels for a 4-parameter model; treat ET values as descriptive. ET90 is above measured positive range (3 to 180). High-dose asymptote is close to a response bound. ET90 bootstrap CI extends outside the measured positive range. ET50 log-scale approximation is very wide or outside the measured range; prefer bootstrap CI. |
| 4PL fitting note | 4PL fitted on positive x levels only using multiple starting values and maxfev=200000. ET50 CI uses a log-scale approximation. Response asymptotes constrained to 0...100. |
| Overall test note | Because a quasibinomial GLM was not fitted in the final workflow, the overall exposure-time effect was evaluated using a fallback chi-square test on infected/non-infected counts, with overdispersion reported separately |

**Supplementary Table S6. Pairwise exposure-time comparisons and effect sizes for Figure 3a.**

Pairwise comparisons are based on two-proportion z-tests. The Holm p-value adjusted for overdispersion is the preferred p-value because overdispersion was high. Cohen's h is the recommended effect size for proportions; Hedges' g is included as an auxiliary standardized mean-difference metric.

| **Comparison** | **Difference (percentage points)** | **Holm p-value adjusted for overdispersion** | **Cohen's h** | **Hedges' g** | **Note** |
| --- | --- | --- | --- | --- | --- |
| 0 s vs 3 s | 4 | 0.607 | 0.403 | 0.571 |  |
| 0 s vs 10 s | 8 | 0.473 | 0.574 | 0.571 |  |
| 0 s vs 30 s | 28 | 0.014 | 1.115 | 3.265 |  |
| 0 s vs 100 s | 44 | <0.001 | 1.451 | 3.359 |  |
| 0 s vs 180 s | 84 | <0.0001 | 2.319 | 6.412 |  |
| 3 s vs 10 s | 4 | 0.607 | 0.171 | 0.255 |  |
| 3 s vs 30 s | 24 | 0.074 | 0.712 | 2.168 |  |
| 3 s vs 100 s | 40 | 0.003 | 1.048 | 2.693 |  |
| 3 s vs 180 s | 80 | <0.0001 | 1.916 | 5.386 |  |
| 10 s vs 30 s | 20 | 0.232 | 0.542 | 1.218 |  |
| 10 s vs 100 s | 36 | 0.014 | 0.877 | 1.877 |  |
| 10 s vs 180 s | 76 | <0.0001 | 1.745 | 3.963 |  |
| 30 s vs 100 s | 16 | 0.607 | 0.335 | 1.022 |  |
| 30 s vs 180 s | 56 | <0.001 | 1.203 | 3.577 |  |
| 100 s vs 180 s | 40 | 0.013 | 0.868 | 2.159 |  |

Abbreviations: ET10, ET50 and ET90, exposure times associated with 10%, 50% and 90% reduction in infection, respectively; SE, standard error; SD, standard deviation; 4PL, four-parameter logistic model.

# Figure 3b: time-response analysis against Aspergillus flavus

**Supplementary Table S7. Descriptive statistics of time-dependent antifungal efficacy of 50 mM PNA against Aspergillus flavus inoculated onto maize seeds.**

Wilson confidence intervals are based on pooled infected/non-infected seed counts within each exposure time. Each replicate consisted of 100 seeds.

| **PNA exposure time** | **Replicates (n)** | **Mean infected seeds (%)** | **SD** | **SE** | **Median** | **Min** | **Max** | **Infected seeds (count)** | **Total seeds assessed** | **Infected seeds (%) from counts** | **Wilson 95% CI** |
| --- | --- | --- | --- | --- | --- | --- | --- | --- | --- | --- | --- |
| 0 s | 5 | 100 | 0 | 0 | 100 | 100 | 100 | 500 | 500 | 100 | 99.24 to 100.0 |
| 3 s | 5 | 80 | 24.49 | 10.95 | 80 | 40 | 100 | 400 | 500 | 80 | 76.27 to 83.27 |
| 10 s | 5 | 68 | 17.89 | 8 | 80 | 40 | 80 | 340 | 500 | 68 | 63.79 to 71.94 |
| 30 s | 5 | 67 | 9.75 | 4.36 | 60 | 60 | 80 | 335 | 500 | 67 | 62.76 to 70.98 |
| 100 s | 5 | 12 | 17.89 | 8 | 0 | 0 | 40 | 60 | 500 | 12 | 9.44 to 15.14 |
| 180 s | 5 | 12 | 10.95 | 4.9 | 20 | 0 | 20 | 60 | 500 | 12 | 9.44 to 15.14 |

**Supplementary Table S8. Overall test, overdispersion and 4PL time-response model parameters for Figure 3b.**

ET values are exposure times required to reduce infection by the stated percentage. Confidence intervals were estimated by bootstrap percentile intervals. Because overdispersion was high, pairwise quasi-adjusted p-values are preferred for interpretation.

| **Parameter** | **Value** |
| --- | --- |
| Analysis type | binomial_count |
| Reported metric | ET |
| Target levels (%) | 10, 50, 90 |
| Primary model | fallback chi-square test: infected/noninfected counts by group |
| Overall test statistic | 1352.4 |
| Overall test df | 5.0000 |
| Overall test p-value | <0.0001 |
| Overdispersion (Pearson chi-square/df) | 16.36 |
| ET10 (s) | 29.32 |
| ET10 95% bootstrap CI, lower (s) | 4.5052 |
| ET10 95% bootstrap CI, upper (s) | 76.50 |
| ET50 (s) | 42.33 |
| ET50 95% bootstrap CI, lower (s) | 32.35 |
| ET50 95% bootstrap CI, upper (s) | 87.50 |
| ET50 log-scale CI status | unstable; prefer bootstrap CI |
| ET90 (s) | 61.13 |
| ET90 95% bootstrap CI, lower (s) | 37.29 |
| ET90 95% bootstrap CI, upper (s) | 298.8 |
| Successful bootstrap fits | 195.0 |
| Bootstrap iterations | 200.0 |
| 4PL low-time asymptote (%) | 74.01 |
| 4PL high-time asymptote (%) | 11.81 |
| 4PL Hill slope | 5.9800 |
| 4PL RMSE on group means | 3.7929 |
| 4PL reliability | use with caution |
| 4PL warnings | Only 5 positive x levels for a 4-parameter model; treat ET values as descriptive. ET90 bootstrap CI extends outside the measured positive range. ET50 log-scale approximation is very wide or outside the measured range; prefer bootstrap CI. |
| 4PL fitting note | 4PL fitted on positive x levels only using multiple starting values and maxfev=200000. ET50 CI uses a log-scale approximation. Response asymptotes constrained to 0...100. |
| Overall test note | Because a quasibinomial GLM was not fitted in the final workflow, the overall exposure-time effect was evaluated using a fallback chi-square test on infected/non-infected counts, with overdispersion reported separately |

**Supplementary Table S9. Pairwise exposure-time comparisons and effect sizes for Figure 3b.**

Pairwise comparisons are based on two-proportion z-tests. The Holm p-value adjusted for overdispersion is the preferred p-value because overdispersion was high. Cohen's h is the recommended effect size for proportions; Hedges' g is included as an auxiliary standardized mean-difference metric.

| **Comparison** | **Difference (percentage points)** | **Holm p-value adjusted for overdispersion** | **Cohen's h** | **Hedges' g** | **Note** |
| --- | --- | --- | --- | --- | --- |
| 0 s vs 3 s | 20 | 0.046 | 0.927 | 1.043 |  |
| 0 s vs 10 s | 32 | 0.004 | 1.203 | 2.285 |  |
| 0 s vs 30 s | 33 | 0.004 | 1.224 | 4.325 |  |
| 0 s vs 100 s | 88 | <0.0001 | 2.434 | 6.284 |  |
| 0 s vs 180 s | 88 | <0.0001 | 2.434 | 10.26 |  |
| 3 s vs 10 s | 12 | 0.998 | 0.275 | 0.505 |  |
| 3 s vs 30 s | 13 | 0.998 | 0.297 | 0.63 |  |
| 3 s vs 100 s | 68 | <0.0001 | 1.507 | 2.864 |  |
| 3 s vs 180 s | 68 | <0.0001 | 1.507 | 3.237 |  |
| 10 s vs 30 s | 1 | 1.000 | 0.021 | 0.063 |  |
| 10 s vs 100 s | 56 | <0.0001 | 1.232 | 2.828 |  |
| 10 s vs 180 s | 56 | <0.0001 | 1.232 | 3.41 |  |
| 30 s vs 100 s | 55 | <0.0001 | 1.21 | 3.449 |  |
| 30 s vs 180 s | 55 | <0.0001 | 1.21 | 4.791 |  |
| 100 s vs 180 s | 0 | 1.000 | 0 | 0.0 |  |

Abbreviations: ET10, ET50 and ET90, exposure times associated with 10%, 50% and 90% reduction in infection, respectively; SE, standard error; SD, standard deviation; 4PL, four-parameter logistic model.

# Figure 4a: Fusarium fujikuroi, PNA exposure time and plasma pre-treatment

**Supplementary Table S10. Descriptive statistics by PNA exposure time and plasma pre-treatment for Fusarium fujikuroi.**

Wilson confidence intervals are based on pooled infected/non-infected seed counts within each time x treatment group. Each replicate consisted of 100 seeds.

| **Treatment group** | **PNA exposure time (s)** | **Replicates (n)** | **Mean infected seeds (%)** | **SD** | **SE** | **Median** | **Min** | **Max** | **Infected seeds (count)** | **Total seeds assessed** | **Infected seeds (%) from counts** | **Wilson 95% CI** |
| --- | --- | --- | --- | --- | --- | --- | --- | --- | --- | --- | --- | --- |
| No plasma | 0 | 5 | 100 | 0 | 0 | 100 | 100 | 100 | 500 | 500 | 100 | 99.24 to 100 |
| No plasma | 3 | 5 | 68 | 30.33 | 13.56 | 80 | 20 | 100 | 340 | 500 | 68 | 63.79 to 71.94 |
| No plasma | 10 | 5 | 64 | 26.08 | 11.66 | 80 | 20 | 80 | 320 | 500 | 64 | 59.70 to 68.09 |
| No plasma | 30 | 5 | 44 | 26.08 | 11.66 | 60 | 0 | 60 | 220 | 500 | 44 | 39.71 to 48.38 |
| No plasma | 100 | 5 | 36 | 26.08 | 11.66 | 40 | 0 | 60 | 180 | 500 | 36 | 31.91 to 40.30 |
| No plasma | 180 | 5 | 28 | 17.89 | 8 | 40 | 0 | 40 | 140 | 500 | 28 | 24.24 to 32.09 |
| No plasma | 300 | 5 | 0 | 0 | 0 | 0 | 0 | 0 | 0 | 500 | 0 | 0.00 to 0.76 |
| Plasma pre-treatment | 0 | 5 | 100 | 0 | 0 | 100 | 100 | 100 | 500 | 500 | 100 | 99.24 to 100 |
| Plasma pre-treatment | 3 | 5 | 60 | 20 | 8.94 | 60 | 40 | 80 | 300 | 500 | 60 | 55.65 to 64.20 |
| Plasma pre-treatment | 10 | 5 | 56 | 26.08 | 11.66 | 60 | 20 | 80 | 280 | 500 | 56 | 51.62 to 60.29 |
| Plasma pre-treatment | 30 | 5 | 28 | 33.47 | 14.97 | 20 | 0 | 80 | 140 | 500 | 28 | 24.24 to 32.09 |
| Plasma pre-treatment | 100 | 5 | 28 | 17.89 | 8 | 40 | 0 | 40 | 140 | 500 | 28 | 24.24 to 32.09 |
| Plasma pre-treatment | 180 | 5 | 24 | 16.73 | 7.48 | 20 | 0 | 40 | 120 | 500 | 24 | 20.46 to 27.93 |
| Plasma pre-treatment | 300 | 5 | 0 | 0 | 0 | 0 | 0 | 0 | 0 | 500 | 0 | 0.00 to 0.76 |

**Supplementary Table S11. Overall test, overdispersion and group-specific 4PL time-response model parameters for Fusarium fujikuroi.**

ET values are exposure times required to reduce infection by the stated percentage. Confidence intervals were estimated by bootstrap percentile intervals. ET90 estimates were outside the observed time range and should therefore be interpreted as extrapolated model descriptors.

| **Section** | **Parameter** | **No plasma** | **Plasma pre-treatment** | **Overall value / note** |
| --- | --- | --- | --- | --- |
| Overall test | Analysis type | binomial_count |  |  |
| Overall test | Reported metric | ET |  |  |
| Overall test | Target levels (%) | 10, 50, 90 |  |  |
| Overall test | Group-time-cell test | fallback chi-square test: infected/non-infected counts by group-time cell |  |  |
| Overall test | Overall test statistic | 2566.1 |  |  |
| Overall test | Overall test df | 13 |  |  |
| Overall test | Overall test p-value | <0.0001 |  |  |
| Diagnostics | Overdispersion (Pearson chi-square/df) | 19.7 |  |  |
| 4PL model | ET10 (s) | 10.84 | 1.878 |  |
| 4PL model | ET10 95% bootstrap CI, lower (s) | 0.297 | 0.053 |  |
| 4PL model | ET10 95% bootstrap CI, upper (s) | 157.9 | 78.17 |  |
| 4PL model | ET50 (s) | 79.64 | 33.51 |  |
| 4PL model | ET50 95% bootstrap CI, lower (s) | 11.45 | 5.412 |  |
| 4PL model | ET50 95% bootstrap CI, upper (s) | 189.3 | 162.7 |  |
| 4PL model | ET90 (s) | 585.3 | 597.9 |  |
| 4PL model | ET90 95% bootstrap CI, lower (s) | 38.03 | 11.91 |  |
| 4PL model | ET90 95% bootstrap CI, upper (s) | 1252.0 | 1153.3 |  |
| 4PL model | Low-time asymptote (%) | 68.58 | 71 |  |
| 4PL model | High-time asymptote (%) | 0 | 0 |  |
| 4PL model | Hill slope | 1.102 | 0.763 |  |
| 4PL model | RSS | 324.6 | 351.6 |  |
| 4PL model | RMSE | 7.355 | 7.655 |  |
| 4PL model | Successful bootstrap fits | 200 | 200 |  |
| 4PL model | Bootstrap iterations | 200 | 200 |  |
| 4PL fitting note | Model fitting |  |  | 4PL models were fitted separately for each treatment group on positive time levels only. The 0 s controls were shown at artificial offset positions for log-scale visualisation only and were not used for log-scale fitting. ET90 estimates were outside the observed time range and should be interpreted as extrapolated model descriptors. |

**Supplementary Table S12. Planned plasma versus no-plasma comparisons at each exposure time for Fusarium fujikuroi.**

Differences are calculated as no plasma minus plasma, so positive values indicate lower infection after plasma pre-treatment. Quasi-adjusted p-values account for high overdispersion and should be interpreted preferentially for binomial count data. Cohen's h is the preferred effect size for proportions; Hedges' g is included as an auxiliary standardized mean-difference metric.

| **Time (s)** | **No plasma mean infection (%)** | **Plasma mean infection (%)** | **Difference (percentage points)** | **Nominal p-value** | **Holm p-value** | **Quasi-adjusted p-value** | **Holm quasi-adjusted p-value** | **Cohen's h** | **Hedges g** |
| --- | --- | --- | --- | --- | --- | --- | --- | --- | --- |
| 0 | 100 | 100 | 0 | 1.000 | 1.000 | 1.000 | 1.000 | 0 | 0 |
| 3 | 68 | 60 | 8 | 0.008 | 0.042 | 0.553 | 1.000 | 0.167 | 0.281 |
| 10 | 64 | 56 | 8 | 0.010 | 0.042 | 0.561 | 1.000 | 0.164 | 0.277 |
| 30 | 44 | 28 | 16 | <0.0001 | <0.0001 | 0.235 | 1.000 | 0.335 | 0.482 |
| 100 | 36 | 28 | 8 | 0.007 | 0.040 | 0.541 | 1.000 | 0.172 | 0.323 |
| 180 | 28 | 24 | 4 | 0.149 | 0.448 | 0.745 | 1.000 | 0.091 | 0.209 |
| 300 | 0 | 0 | 0 | 1.000 | 1.000 | 1.000 | 1.000 | 0 | 0 |

Abbreviations: CP, cold plasma; ET10, ET50 and ET90, exposure times associated with 10%, 50% and 90% reduction in infection, respectively; SE, standard error; SD, standard deviation; 4PL, four-parameter logistic model; RSS, residual sum of squares; RMSE, root mean squared error.

# Figure 4b: Aspergillus flavus, PNA exposure time and plasma pre-treatment

**Supplementary Table S13. Descriptive statistics by PNA exposure time and plasma pre-treatment for Aspergillus flavus.**

Wilson confidence intervals are based on pooled infected/non-infected seed counts within each time x treatment group. Each replicate consisted of 100 seeds.

| **Treatment group** | **PNA exposure time (s)** | **Replicates (n)** | **Mean infected seeds (%)** | **SD** | **SE** | **Median** | **Min** | **Max** | **Infected seeds (count)** | **Total seeds assessed** | **Infected seeds (%) from counts** | **Wilson 95% CI** |
| --- | --- | --- | --- | --- | --- | --- | --- | --- | --- | --- | --- | --- |
| No plasma | 0 | 5 | 100 | 0 | 0 | 100 | 100 | 100 | 500 | 500 | 100 | 99.24 to 100 |
| No plasma | 3 | 5 | 48 | 30.33 | 13.56 | 40 | 20 | 80 | 240 | 500 | 48 | 43.65 to 52.38 |
| No plasma | 10 | 5 | 48 | 22.8 | 10.2 | 40 | 20 | 80 | 240 | 500 | 48 | 43.65 to 52.38 |
| No plasma | 30 | 5 | 28 | 10.95 | 4.9 | 20 | 20 | 40 | 140 | 500 | 28 | 24.24 to 32.09 |
| No plasma | 100 | 5 | 28 | 17.89 | 8 | 40 | 0 | 40 | 140 | 500 | 28 | 24.24 to 32.09 |
| No plasma | 180 | 5 | 16 | 16.73 | 7.48 | 20 | 0 | 40 | 80 | 500 | 16 | 13.05 to 19.47 |
| No plasma | 300 | 5 | 0 | 0 | 0 | 0 | 0 | 0 | 0 | 500 | 0 | 0.00 to 0.76 |
| Plasma pre-treatment | 0 | 5 | 100 | 0 | 0 | 100 | 100 | 100 | 500 | 500 | 100 | 99.24 to 100 |
| Plasma pre-treatment | 3 | 5 | 36 | 26.08 | 11.66 | 40 | 0 | 60 | 180 | 500 | 36 | 31.91 to 40.30 |
| Plasma pre-treatment | 10 | 5 | 28 | 10.95 | 4.9 | 20 | 20 | 40 | 140 | 500 | 28 | 24.24 to 32.09 |
| Plasma pre-treatment | 30 | 5 | 16 | 8.94 | 4 | 20 | 0 | 20 | 80 | 500 | 16 | 13.05 to 19.47 |
| Plasma pre-treatment | 100 | 5 | 8 | 10.95 | 4.9 | 0 | 0 | 20 | 40 | 500 | 8 | 5.93 to 10.71 |
| Plasma pre-treatment | 180 | 5 | 8 | 10.95 | 4.9 | 0 | 0 | 20 | 40 | 500 | 8 | 5.93 to 10.71 |
| Plasma pre-treatment | 300 | 5 | 0 | 0 | 0 | 0 | 0 | 0 | 0 | 500 | 0 | 0.00 to 0.76 |

**Supplementary Table S14. Overall test, overdispersion and group-specific 4PL time-response model parameters for Aspergillus flavus.**

ET values are exposure times required to reduce infection by the stated percentage. Confidence intervals were estimated by bootstrap percentile intervals. The no-plasma ET90 estimate was outside the observed time range; the plasma ET90 confidence interval was wide and should be interpreted cautiously as a model descriptor.

| **Section** | **Parameter** | **No plasma** | **Plasma pre-treatment** | **Overall value / note** |
| --- | --- | --- | --- | --- |
| Overall test | Analysis type | binomial_count |  |  |
| Overall test | Reported metric | ET |  |  |
| Overall test | Target levels (%) | 10, 50, 90 |  |  |
| Overall test | Group-time-cell test | fallback chi-square test: infected/non-infected counts by group-time cell |  |  |
| Overall test | Overall test statistic | 3050.3 |  |  |
| Overall test | Overall test df | 13 |  |  |
| Overall test | Overall test p-value | <0.0001 |  |  |
| Diagnostics | Overdispersion (Pearson chi-square/df) | 12.46 |  |  |
| 4PL model | ET10 (s) | 8.699 | 1.658 |  |
| 4PL model | ET10 95% bootstrap CI, lower (s) | 0.117 | 0.034 |  |
| 4PL model | ET10 95% bootstrap CI, upper (s) | 167.3 | 35.39 |  |
| 4PL model | ET50 (s) | 68.81 | 18.73 |  |
| 4PL model | ET50 95% bootstrap CI, lower (s) | 4.687 | 1.502 |  |
| 4PL model | ET50 95% bootstrap CI, upper (s) | 189.4 | 94.94 |  |
| 4PL model | ET90 (s) | 544.3 | 211.6 |  |
| 4PL model | ET90 95% bootstrap CI, lower (s) | 32.86 | 23.59 |  |
| 4PL model | ET90 95% bootstrap CI, upper (s) | 830.3 | 589.4 |  |
| 4PL model | Low-time asymptote (%) | 50.09 | 43 |  |
| 4PL model | High-time asymptote (%) | 0 | 0 |  |
| 4PL model | Hill slope | 1.062 | 0.906 |  |
| 4PL model | RSS | 213 | 21.3 |  |
| 4PL model | RMSE | 5.957 | 1.885 |  |
| 4PL model | Successful bootstrap fits | 200 | 200 |  |
| 4PL model | Bootstrap iterations | 200 | 200 |  |
| 4PL fitting note | Model fitting |  |  | 4PL models were fitted separately for each treatment group on positive time levels only. The 0 s controls were shown at artificial offset positions for log-scale visualisation only and were not used for log-scale fitting. The no-plasma ET90 estimate was outside the observed time range; the plasma ET90 confidence interval was wide and should be interpreted cautiously as a model descriptor. |

**Supplementary Table S15. Planned plasma versus no-plasma comparisons at each exposure time for Aspergillus flavus.**

Differences are calculated as no plasma minus plasma, so positive values indicate lower infection after plasma pre-treatment. Quasi-adjusted p-values account for high overdispersion and should be interpreted preferentially for binomial count data. Cohen's h is the preferred effect size for proportions; Hedges' g is included as an auxiliary standardized mean-difference metric.

| **Time (s)** | **No plasma mean infection (%)** | **Plasma mean infection (%)** | **Difference (percentage points)** | **Nominal p-value** | **Holm p-value** | **Quasi-adjusted p-value** | **Holm quasi-adjusted p-value** | **Cohen's h** | **Hedges g** |
| --- | --- | --- | --- | --- | --- | --- | --- | --- | --- |
| 0 | 100 | 100 | 0 | 1.000 | 1.000 | 1.000 | 1.000 | 0 | 0 |
| 3 | 48 | 36 | 12 | <0.001 | <0.001 | 0.276 | 1.000 | 0.244 | 0.383 |
| 10 | 48 | 28 | 20 | <0.0001 | <0.0001 | 0.065 | 0.389 | 0.416 | 1.01 |
| 30 | 28 | 16 | 12 | <0.0001 | <0.0001 | 0.194 | 0.972 | 0.292 | 1.084 |
| 100 | 28 | 8 | 20 | <0.0001 | <0.0001 | 0.020 | 0.138 | 0.542 | 1.218 |
| 180 | 16 | 8 | 8 | <0.0001 | <0.001 | 0.270 | 1.000 | 0.25 | 0.511 |
| 300 | 0 | 0 | 0 | 1.000 | 1.000 | 1.000 | 1.000 | 0 | 0 |

Abbreviations: CP, cold plasma; ET10, ET50 and ET90, exposure times associated with 10%, 50% and 90% reduction in infection, respectively; SE, standard error; SD, standard deviation; 4PL, four-parameter logistic model; RMSE, root mean squared error.

# Germination analysis

**Supplementary Table S16. Descriptive statistics for maize seed germination after PNA and plasma-related treatments.**

Values are summarized from replicate-level germination percentages. C, untreated control; P, hydrogen peroxide; CP, cold plasma treatment.

| **Treatment** | **Replicates (n)** | **Mean germination (%)** | **SD** | **SE** | **Median** | **Q1** | **Q3** | **Min** | **Max** |
| --- | --- | --- | --- | --- | --- | --- | --- | --- | --- |
| C | 4 | 90.22 | 8.09 | 4.04 | 89.52 | 84.74 | 95 | 81.82 | 100 |
| 100 mM PNA | 3 | 88.38 | 4.37 | 2.53 | 90.91 | 87.12 | 90.91 | 83.33 | 90.91 |
| 200 mM PNA | 3 | 88.64 | 12.65 | 7.31 | 90.91 | 82.95 | 95.45 | 75 | 100 |
| 300 mM PNA | 3 | 82.58 | 7.98 | 4.61 | 81.82 | 78.41 | 86.36 | 75 | 90.91 |
| 400 mM PNA | 3 | 94.44 | 9.62 | 5.56 | 100 | 91.67 | 100 | 83.33 | 100 |
| P | 3 | 94.19 | 5.04 | 2.91 | 91.67 | 91.29 | 95.83 | 90.91 | 100 |
| CP | 4 | 93.33 | 9.43 | 4.71 | 96.67 | 90 | 100 | 80 | 100 |

**Supplementary Table S17. Assumption checks, global test and practical relevance thresholds for germination.**

Normality was assessed by Shapiro-Wilk tests within groups and variance homogeneity by median-centred Levene test. Because normality was not confirmed in all groups, the Kruskal-Wallis test was selected for the global treatment comparison.

| **Section** | **Parameter** | **Value** | **Interpretation** |
| --- | --- | --- | --- |
| Normality | C Shapiro-Wilk p-value | 0.812 | Normality not rejected |
| Normality | 100 mM PNA Shapiro-Wilk p-value | <0.0001 | Normality not confirmed |
| Normality | 200 mM PNA Shapiro-Wilk p-value | 0.702 | Normality not rejected |
| Normality | 300 mM PNA Shapiro-Wilk p-value | 0.843 | Normality not rejected |
| Normality | 400 mM PNA Shapiro-Wilk p-value | <0.0001 | Normality not confirmed |
| Normality | P Shapiro-Wilk p-value | 0.144 | Normality not rejected |
| Normality | CP Shapiro-Wilk p-value | 0.161 | Normality not rejected |
| Variance homogeneity | Levene test p-value | 0.902 | Variance heterogeneity not detected |
| Selected global test | Kruskal-Wallis H | 5.164 | Non-parametric omnibus test |
| Selected global test | Kruskal-Wallis p-value | 0.523 | No statistically significant overall treatment effect at alpha = 0.05 |
| Global effect size | Eta squared (descriptive) | 0.2188 | Descriptive ANOVA-type effect size only; not used as the inferential result |
| Global effect size | Epsilon squared | 0 | Negligible Kruskal-Wallis effect size |
| Practical relevance threshold | SESOI | 4.094 percentage points | CV-based threshold: 0.5 x mean within-group CV x overall mean |

**Supplementary Table S18. Pairwise post-hoc comparisons, Hedges g and practical relevance flags for germination.**

Pairwise comparisons used Mann-Whitney tests with Holm correction. Mean differences are first group minus second group; positive values indicate higher germination in the first group. Differences exceeding the SESOI are flagged as biologically relevant but statistically uncertain when the Holm-adjusted p-value is not significant.

| **Comparison** | **Delta mean (percentage points)** | **Hedges g** | **Magnitude** | **Holm-adjusted p-value** | **Significant** | **Exceeds SESOI** | **Note** |
| --- | --- | --- | --- | --- | --- | --- | --- |
| C vs 100 mM PNA | 1.83 | 0.225 | small | 1 | no | No |  |
| C vs 200 mM PNA | 1.58 | 0.131 | negligible | 1 | no | No |  |
| C vs 300 mM PNA | 7.64 | 0.8 | moderate | 1 | no | Yes | Exceeds SESOI; statistically uncertain |
| C vs 400 mM PNA | -4.23 | -0.408 | small | 1 | no | Yes | Exceeds SESOI; statistically uncertain |
| C vs P | -3.98 | -0.476 | small | 1 | no | No |  |
| C vs CP | -3.12 | -0.309 | small | 1 | no | No |  |
| 100 mM PNA vs 200 mM PNA | -0.25 | -0.021 | negligible | 1 | no | No |  |
| 100 mM PNA vs 300 mM PNA | 5.81 | 0.722 | moderate | 1 | no | Yes | Exceeds SESOI; statistically uncertain |
| 100 mM PNA vs 400 mM PNA | -6.06 | -0.649 | moderate | 1 | no | Yes | Exceeds SESOI; statistically uncertain |
| 100 mM PNA vs P | -5.81 | -0.984 | large | 1 | no | Yes | Exceeds SESOI; statistically uncertain |
| 100 mM PNA vs CP | -4.95 | -0.534 | moderate | 1 | no | Yes | Exceeds SESOI; statistically uncertain |
| 200 mM PNA vs 300 mM PNA | 6.06 | 0.458 | small | 1 | no | Yes | Exceeds SESOI; statistically uncertain |
| 200 mM PNA vs 400 mM PNA | -5.81 | -0.413 | small | 1 | no | Yes | Exceeds SESOI; statistically uncertain |
| 200 mM PNA vs P | -5.56 | -0.461 | small | 1 | no | Yes | Exceeds SESOI; statistically uncertain |
| 200 mM PNA vs CP | -4.7 | -0.365 | small | 1 | no | Yes | Exceeds SESOI; statistically uncertain |
| 300 mM PNA vs 400 mM PNA | -11.87 | -1.07 | large | 1 | no | Yes | Exceeds SESOI; statistically uncertain |
| 300 mM PNA vs P | -11.62 | -1.39 | large | 1 | no | Yes | Exceeds SESOI; statistically uncertain |
| 300 mM PNA vs CP | -10.76 | -1.02 | large | 1 | no | Yes | Exceeds SESOI; statistically uncertain |
| 400 mM PNA vs P | 0.25 | 0.026 | negligible | 1 | no | No |  |
| 400 mM PNA vs CP | 1.11 | 0.098 | negligible | 1 | no | No |  |
| P vs CP | 0.86 | 0.091 | negligible | 1 | no | No |  |

**Supplementary Table S19. Equivalence testing versus the untreated control for germination.**

TOST equivalence tests were performed against the untreated control (C) using equivalence bounds of +/- SESOI on the original germination-percentage scale. Equivalence requires both one-sided p-values to be below alpha; otherwise similarity to control remains inconclusive rather than proven.

| **Comparison versus control** | **TOST lower p-value** | **TOST upper p-value** | **Conclusion** |
| --- | --- | --- | --- |
| 100 mM PNA vs C | 0.328 | 0.136 | not proven equivalent to control |
| 200 mM PNA vs C | 0.391 | 0.271 | not proven equivalent to control |
| 300 mM PNA vs C | 0.705 | 0.060 | not proven equivalent to control |
| 400 mM PNA vs C | 0.147 | 0.507 | not proven equivalent to control |
| P vs C | 0.084 | 0.491 | not proven equivalent to control |
| CP vs C | 0.145 | 0.440 | not proven equivalent to control |

Abbreviations: CP, cold plasma treatment; P, hydrogen peroxide treatment; SD, standard deviation; SE, standard error; SESOI, smallest effect size of interest; TOST, two one-sided tests.Compact letter display was not applied because the global test was not statistically significant at alpha = 0.05.
